# Outcome prediction for patients assessed by the medical emergency team: a retrospective cohort study

**DOI:** 10.1186/s12873-022-00739-w

**Published:** 2022-12-09

**Authors:** Anna Adielsson, Christian Danielsson, Pontus Forkman, Thomas Karlsson, Linda Pettersson, Johan Herlitz, Stefan Lundin

**Affiliations:** 1 Department of Anaesthesiology and Intensive Care Medicine, Sahlgrenska University Hospital, Gothenburg, Sweden; 2 Department of Clinical Pathology, Sahlgrenska University Hospital, Gothenburg, Sweden; 3 Department of Adult Psychiatry, Mora Hospital, Mora, Sweden; 4 Health Metrics at Sahlgrenska Academy, University of Gothenburg, Gothenburg, Sweden; 5 Center for Clinical Research Dalarna, Department of Public Health and Caring Sciences, Uppsala University, Uppsala, Sweden; 6 The Center for Pre-Hospital Research in Western Sweden, University College of Borås, Borås, Sweden

**Keywords:** Medical emergency team, Rapid response team, Risk score, Outcome, Mortality

## Abstract

**Background:**

Medical emergency teams (METs) have been implemented to reduce hospital mortality by the early recognition and treatment of potentially life-threatening conditions. The objective of this study was to establish a clinically useful association between clinical variables and mortality risk, among patients assessed by the MET, and further to design an easy-to-use risk score for the prediction of death within 30 days.

**Methods:**

Observational retrospective register study in a tertiary university hospital in Sweden, comprising 2,601 patients, assessed by the MET from 2010 to 2015. Patient registry data at the time of MET assessment was analysed from an epidemiological perspective, using univariable and multivariable analyses with death within 30 days as the outcome variable. Predictors of outcome were defined from age, gender, type of ward for admittance, previous medical history, acute medical condition, vital parameters and laboratory biomarkers. Identified factors independently associated with mortality were then used to develop a prognostic risk score for mortality.

**Results:**

The overall 30-day mortality was high (29.0%). We identified thirteen factors independently associated with 30-day mortality concerning; age, type of ward for admittance, vital parameters, laboratory biomarkers, previous medical history and acute medical condition. A MET risk score for mortality based on the impact of these individual thirteen factors in the model yielded a median (range) AUC of 0.780 (0.774–0.785) with good calibration. When corrected for optimism by internal validation, the score yielded a median (range) AUC of 0.768 (0.762–0.773).

**Conclusions:**

Among clinical variables available at the time of MET assessment, thirteen factors were found to be independently associated with 30-day mortality. By applying a simple risk scoring system based on these individual factors, patients at higher risk of dying within 30 days after the MET assessment may be identified and treated earlier in the process.

**Supplementary Information:**

The online version contains supplementary material available at 10.1186/s12873-022-00739-w.

## Background

Over the years, healthcare has evolved rapidly with increasingly older and more ill patients being attended to, resulting in immense demand for clinical resources. Rapid response systems, including medical emergency teams (MET), provide unstable patients in general wards with access to critical care expertise when early signs of clinical deterioration are recognised [1–3]. Despite this, intensive care resources are insufficient with critically ill patient demand being greater than available bed supply [4, 5]. Currently, no dedicated tool is available to identify patients at risk or to refine the selection of patients who would benefit most from admission to the intensive care unit (ICU), in terms of survival. It would, therefore, be desirable to refine the selection of patients who would benefit most from admission to the intensive care unit (ICU).

The objective of this study was to establish a clinically useful association between clinical variables and mortality risk, among patients assessed by the MET. Early identification and risk stratification of an impending patient crisis are valuable in the guidance of further therapeutic efforts and adaptation to available resources [6, 7].

We hypothesised that it is possible to identify factors at the time of MET assessment associated with an increased mortality risk during the subsequent 30 days. Potential risk factors could then be considered more systematically as a decision basis when prioritising and optimising the chain of care.

The outcome variable was 30-day mortality, and the potential predictors were defined from age, gender, type of ward for admittance, previous medical history, acute medical condition, vital parameters and laboratory biomarkers.

## Methods

### Settings

The study was performed at Sahlgrenska University Hospital in Gothenburg, Sweden, which provides specialised care and is the trauma referral centre for the entire region of 1.7 million inhabitants. The study hospital has some 700 beds available for close to 50,000 admissions and 18,000 surgical procedures each year**.** The MET service was introduced in 2005. Since 2007, the MET service has operated at full scale on all nursing wards, except for thoracic surgery wards, receiving approximately 600 consultations annually. A breakdown of the number of MET activations versus hospital admissions, i.e. the MET dose, is reported, including the annual distribution of ICU admissions and outcome (Additional file 1).

### MET system

The MET system is designed to be activated by ward staff in patients with abnormal vital parameters, or when any of the staff feels worried about the patient [8]. In the event of an immediate life-threatening condition, the cardiac arrest team (CAT) should be alerted.

During the study period, the MET service was available 24 h/day, all week. The MET included an intensive care specialist at consultant level during the day and an intensive care resident physician at night, plus an intensive care nurse. The MET system utilised a single-parameter track-and-trigger system, with the following activation criteria:

- Saturation < 90% despite oxygen administration
- Respiratory rate < 8 or > 30 breaths/minute
- Systolic blood pressure < 90 mmHg
- Heart rate < 40 or > 130 beats/minute
- Decreased level of consciousness
- Serious concern about the patient's condition

Until September 2013, 'threatened airway' was included as a criterion. However, due to the seriousness of this condition, it was removed to be handled by the CAT instead. During the time of the study, individually tailored ordinations for checking vital parameters were applied. Given the diversity of ward patients, a universal praxis was refrained from. Depending on clinical findings, ward nurses were accredited to independently administer oxygen to patients with hypoxia.

### Study design

The study was a retrospective, observational study of registry data on MET assessed patients, from 1 January 2010 to 31 December 2015. The data was analysed using univariable and multivariable analyses with death within 30 days as the endpoint. Identified factors independently associated with mortality were then used to develop a prognostic risk score for 30-day mortality.

The first step in our analysis was directed at identifying factors associated with 30-day mortality risk among patients assessed by the MET. The second step was directed at designing an easy-to-use risk score for the prediction of death within 30 days.

### Study population

**Fig. 1:**
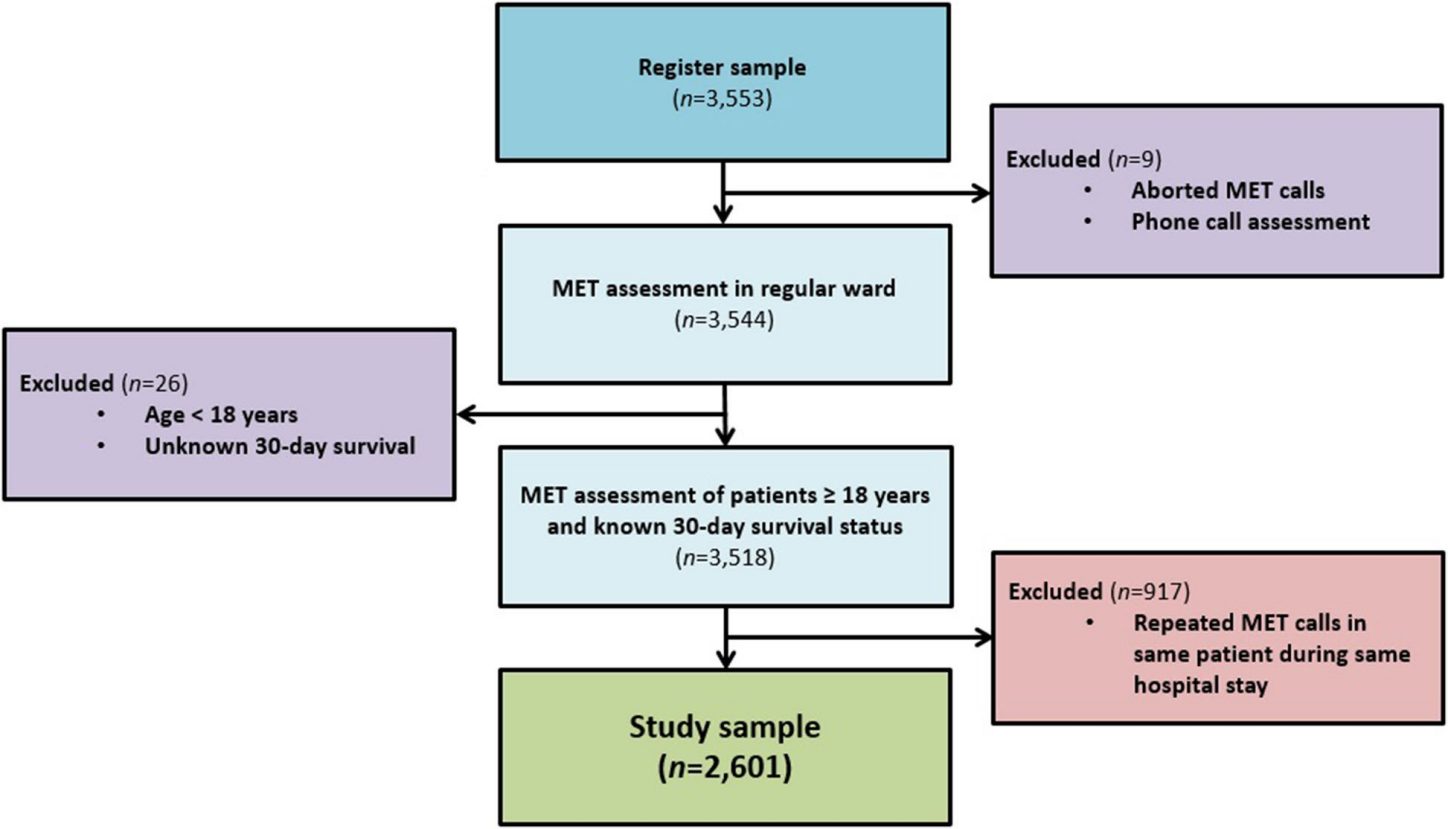
Flow chart of study participants; Register sample of patients where MET was activated while hospitalised in 2010–2015 at Sahlgrenska University Hospital

To be included, patients had to be assessed by the MET on general wards and registered in a standardised protocol. The patient had to be 18 years or older, with known 30-day survival status. In the event of repeated MET assessments, only the first MET assessment during each hospital episode was included in the main study (Fig. 1).

To investigate whether patients requiring additional MET assessments during the same admission period differed from patients requiring only one MET assessment, these two patient groups were compared regarding age, gender and previous medical history (Additional file 2).

### Data collection

Data were collected from the assessment protocol, supplemented with electronic medical records. Baseline characteristics, type of ward for admittance, previous medical history, the reason for MET call, vital parameters at MET arrival, biomarkers from up to 48 h before and six hours after MET activation, acute medical condition, limitation of medical therapy (LOMT), potential ICU admission and primary diagnosis were recorded (Additional file 3). Thirty-day survival status was obtained and confirmed from the Swedish population registry.

### Statistical analysis

The statistical methodology in this study has previously been used for the prediction of poor outcome among early survivors after out-of-hospital cardiac arrest and the development of a risk score [9]. A description of patient characteristics, divided by the outcome of death within 30 days, is shown in Additional files 4, 5, 6  7 and 8. Categorical variables are presented as numbers and percentages, and continuous variables are presented as medians with 10th and 90th percentiles. Multivariable logistic regression was used for age-adjusted *p*-value calculation. Due to missing data, assumed to be missing at random, multiple imputations were used in the multivariable analyses.

For identification of independent predictors of death during the first 30 days, a full model including all variables in Additional files 4, 5, 6, 7 and 8 with age-adjusted *p* < 0.20 and age itself was used. Multivariable logistic regression was performed in all individual imputed datasets and the variable with the highest *p*-value was excluded. This process was reiterated until all variables reached a *p*-value below 0.01. Based on these statistically filtered variables, a prognostic risk score (MET risk score) for the risk of death within 30-days was developed. To adapt the model to clinical work, a procedure similar to the development of the Framingham Risk Score [10] was applied*.* The increase in risk associated with an increase in age by five years, reflected by five times the beta-coefficient for age in the final model, was decided to be equivalent to one point. Points associated with the individual categories of each identified risk factor were then assessed.

The Hosmer–Lemeshow goodness-of-fit test and the area under the ROC curve (AUC) were used to evaluate calibration and discrimination, respectively. The concordance percentage for all imputed datasets was presented. All imputed datasets were used to assess sensitivity, specificity, and positive and negative predicted values.

Two-sided tests were used and *p*-values below 0.01 were regarded as significant. All analyses were performed with SAS version 9.4 for Windows software.

## Results

### Study participants and outcome

In total, 2,601 patients fulfilled the inclusion criteria (Fig. 1). The study population comprised patients 18 to 99 years of age (mean = 65.7, standard deviation 16.8), of which 44.3% were female. Higher age was associated with a significantly higher 30-day mortality. Fewer than half the patients (42.5%) were transferred to the ICU. Overall 30-day mortality was 29.0%. Patients with palliative decisions and LOMT demonstrated a significantly higher 30-day mortality (65.5%), in comparison to patients without any treatment restrictions (21.2%). There were, however, no significant differences in 30-day mortality with regard to gender or level of care (age-adjusted *p* = 0.37 and 0.31, respectively) (Additional file 9).

### Factors associated with mortality

MET assessed patients on geriatric, respiratory medicine and oncology wards had higher 30-day mortality, whereas MET assessed patients on surgical wards had lower 30-day mortality. Previous conditions associated with higher 30-day mortality were cardiac failure, followed by haematological disease, angina pectoris and pulmonary disease. Acute conditions associated with higher 30-day mortality were gastroenteritis, acute coronary syndrome, cardiac failure and renal failure. Laboratory biomarkers associated with higher 30-day mortality were acidosis, hypoxaemia, hyponatraemia, hypernatraemia, hyperkalaemia, hypoglycaemia, elevated serum creatinine and hyperlactataemia. In terms of vital parameters, the most frequent abnormalities were hypoxia and tachypnoea. Patients who presented with these findings also had higher 30-day mortality (Additional file 4, 5, 6, 7 and 8).

### Factors independently associated with mortality

Through multivariable analysis thirteen factors were identified concerning; age, type of ward for admittance, vital parameters, laboratory biomarkers, previous medical history and acute medical condition, all contributing to the prediction of death. Apart from age, factors independently associated with 30-day mortality included; hypoglycaemia, acute renal failure, unconsciousness, haematological disease, hyperlactataemia, and cancer. Other factors also independently associated with 30-day mortality were; liver disease, anaemia, hypoxia, hypoxaemia and respiratory rate. On the contrary, admittance to surgical wards was independently associated with 30-day survival (Table 1).

**Table 1:** Multivariable analysis of predictors of 30-day mortality for patients where MET was activated while hospitalised in 2010–2015 at Sahlgrenska University Hospital, using multiple imputations

*MULTIVARIABLE ANALYSIS USING MULTIPLE IMPUTATIONS*			
	OR (95% CI)	p	**beta-coefficient**
**AGE**	1.044 (1.036,1.052)^a^	< 0.0001	0.0432^a^
**TYPE OF WARD**			
Surgical ward	0.45 (0.36,0.56)	< 0.0001	-0.8001
**VITAL PARAMETERS**			
SpO2	1.62 (1.42,1.86)^b^	< 0.0001	0.4851^b^
RR	1.031 (1.019,1.044)^c^	< 0.0001	0.0305^c^
RLS	1.81 (1.52,2.16)^d^	< 0.0001	0.5936^d^
**BIOMARKERS**			
Ln(pO2)	0.67 (0.51,0.88)^e^	0.004	-0.4037^e^
Haemoglobin < 90 g/l	1.53 (1.16,2.03)	0.003	0.4263
Glucose < 4.2 mmol/l	2.89 (1.32,6.33)	0.008	1.0622
Sqrt(Lactate)	1.78 (1.46,2.17)^f^	< 0.0001	0.5789^f^
**PREVIOUS MEDICAL HISTORY**			
Liver disease	1.70 (1.22,2.39)	0.002	0.5344
Haematological disease	1.81 (1.25,2.63)	0.002	0.5947
Cancer	1.76 (1.42,2.18)	< 0.0001	0.5637
**ACUTE MEDICAL CONDITION**			
Renal failure	1.82 (1.29,2.56)	0.0006	0.5988

755 (29.0%) endpoints of 2,601 patients

*OR* Odds ratio, *CI* Confidence interval, *SpO2* Peripheral capillary oxygen saturation, *RR* Respiratory rate, *RLS* Reaction level scale

^a^ year

^b^ < 90% vs 90–95% vs > 95%

^c^ breath/min

^d^ > 3 vs 2–3 vs 1

^e^ kPa, transformed by the natural logarithm

^f^ mmol/l, square root transformed

### A risk score for mortality after MET assessment: discrimination and performance

**Fig. 2:**
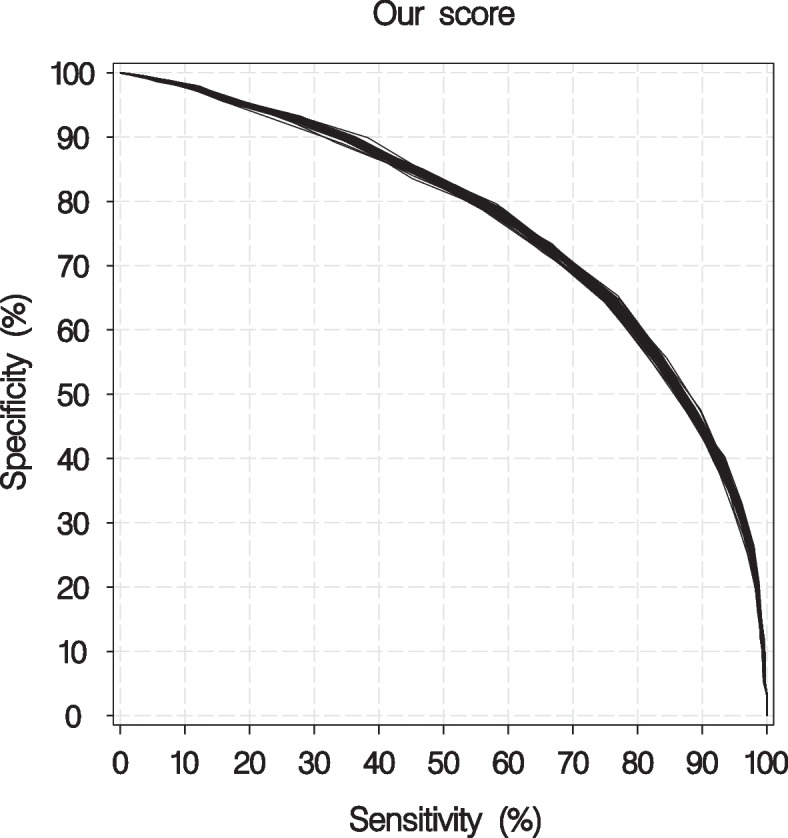
Sensitivity and specificity of our score model. The median AUC for the MET risk score was 0.780 (0.768 corrected to optimism)

The MET risk score for mortality was developed using the final selection of the thirteen factors independently associated with 30-day mortality. The points assigned to different factors are listed (Table 2). The minimum sum of points was -9 and the maximum sum was 49. The performance of the MET score using quartiles as cut-offs is described in Table 3. The median (range) AUC was 0.780 (0.774–0.785) and 0.768 (0.762–0.773) when corrected for optimism by internal validation (Fig. 2). The Hosmer–Lemeshow goodness-of-fit test yielded a *p*-value > 0.05 in all 50 imputed datasets, indicating good calibration. The median concordance percentage was 75.7 (range 75.1–76.3). In patients with a score above 14 points, the sensitivity for 30-day mortality was 75–77%, with a corresponding specificity of 64–65% (Table 3).

**Table 2:** Points assigned to categories of the thirteen independent factors associated with 30-day mortality in the calculation of the MET risk score. The total score ranged from -9 to 49

*RISK SCORE POINTS (-9 to 49)*		
	Categories	Points
Age (years)	< 30	-2
	30–39	0
	40–49	2
	50–59	4
	60–69	6
	70–79	8
	80–89	10
	≥ 90	11
Type of ward	Surgical	-4
	Other	0
SpO2 (%)	> 95	0
	90–95	2
	< 90	4
RR (breaths/min)	< 15	-1
	15–24	0
	25–34	1
	35–44	3
	≥ 45	4
RLS	1	0
	2–3	3
	≥ 4	5
pO2 (kPa)	> 20.0	-1
	10.1–20.0	0
	5.1–10.0	1
	≤ 5.0	2
Haemoglobin (g/l)	≥ 90	0
	< 90	2
Glucose (mmol/l)	≥ 4.2	0
	< 4.2	5
Lactate (mmol/l)	< 1.0	-1
	1.0–1.9	0
	2.0–3.9	1
	4.0–7.9	3
	≥ 8.0	5
Liver disease	No	0
	Yes	2
Haematological disease	No	0
	Yes	3
Cancer	No	0
	Yes	3
Renal failure	No	0
	Yes	3

*SpO2* Peripheral capillary oxygen saturation, *RR* Respiratory rate, *RLS* Reaction level scale

**Table 3:** Discrimination performance of the (-9 to 49) score. The MET risk score is divided into quartiles, where the statistic cut-off (> 14 points) coincides with the second quartile upper limit (i.e. the median)

*DISCRIMINATION PERFORMANCE*			
	>10	>14	>17
n (%)	1957–1985 (75–76)	1213–1242 (47–48)	624–659 (24–25)
Sensitivity (%)	95–96	75–77	45–48
Specificity (%)	31–33	64–65	84–85
PPV (%)	36–37	46–48	53–56
NPV (%)	94–95	86–87	79–80

*PPV* Positive predictive value, *NPV* Negative predictive value

### The occurrence of death over time after MET assessment

**Fig. 3:**
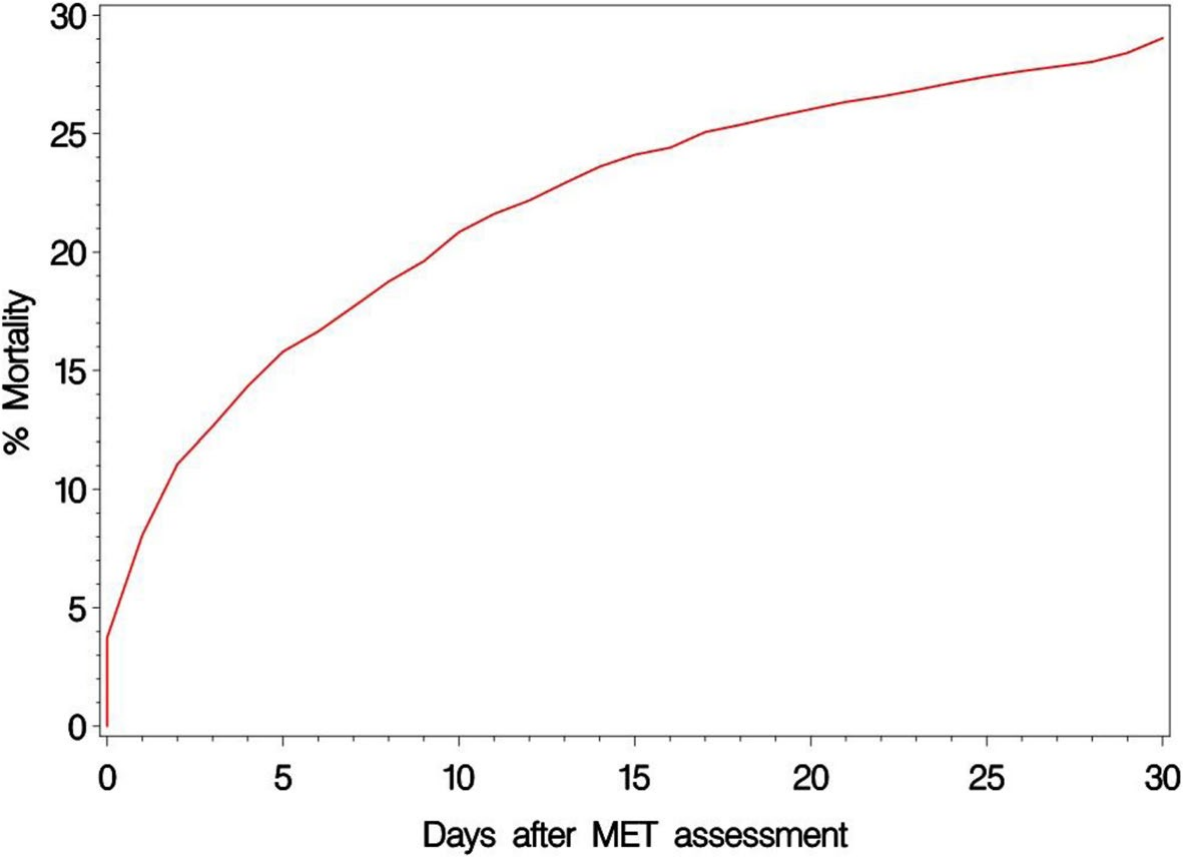
The occurrence of death over time after MET assessment in relation to days after MET assessment while hospitalised in 2010–2015 at Sahlgrenska University Hospital

To illustrate the time of death after MET assessment in more detail, cumulative mortality curves calculated on raw numbers for the thirteen factors independently associated with 30-day mortality (Table 1) are presented (Additional file 10). Overall, approximately half of the deaths occurred within the first four days after the MET assessment (Fig. 3).

## Discussion

The main findings in this study were that overall mortality among patients triggering the MET was high. More than one out of four patients died within 30 days, and the higher the age, the greater the mortality, independent of gender. The medical conditions of patients, such as haematological disease, liver disease, cancer and renal failure, were independently and significantly associated with increased 30-day mortality. The laboratory biomarkers corresponding to the highest mortality risk were hypoglycaemia, hyperlactataemia, anaemia and hypoxaemia. In addition, this study implied the importance of several other factors associated with mortality in clinically deteriorating patients, including abnormal vital parameters such as hypoxia and tachypnoea, level of consciousness and type of ward activating the MET. Overall, thirteen factors independently associated with 30-day mortality were identified, that could be applied for risk stratification and prediction of death within 30 days in MET assessed patients, with acceptable discrimination and performance (median AUC = 0.768 corrected to optimism).

To demonstrate independent risk factors, multivariable analyses were performed. Due to the large quantity of included variables, more than half the study population was excluded from the complete data analysis, as a result of missing data on one or more of these variables (Additional file 11). Therefore, a multivariable analysis using multiple imputations was performed (Table 1). Regardless of the method, the results of both multivariable analyses were consistent with those of the analyses of each variable separately.

The notably high mortality rate in this MET population could be explained by the possibly life-threatening situation of clinical deterioration in combination with advanced age and numerous co-morbidities. As stated in previous studies, MET patients are in the poorest condition among hospitalised patients with high in-hospital and 30-day death rates [1, 11]. Analogously, the type of ward demonstrating by far the highest risk of death among admitted patients was the geriatric wards (Additional file 4). More than half of the clinically deteriorated geriatric patients triggering the MET died within 30 days after assessment. Given the indisputable importance of age in relation to survival, the potentially beneficial contribution of the patient's age as a trigger component in the early warning system cannot be ignored. In this context, it is worth pointing out that although there is a strong correlation between age and survival, it does not necessarily indicate that all elderly patients should be given immediate priority to the ICU. A risk assessment based on the significance of age may be further refined by the inclusion of a frailty index for better adjustment of the age factor to reality [12]. Furthermore, some elderly patients with a particularly high risk of death may be candidates for decisions on LOMT and do not attempt cardiopulmonary resuscitation rather than a higher level of care. Such difficult, but important, decisions in the final stages of life may be facilitated through other decision support tools [13]. However, that kind of reasoning should preferably be processed before alerting the MET.

The association between the type of ward for admittance and mortality reflects the commonly found medical conditions. It appears that medical and surgical wards tend to utilise the MET to about the same extent, although the difference in outcome was striking in our study. The overall 30-day mortality on medical wards was almost twice as high as that on surgical wards. Consequently, the patient's place of care may be indicative of the end of the course, in terms of mortality, as medical ward patients proved to have a significantly increased risk of death. The difference in 30-day mortality between surgical and medical ward patients could be explained in part by the fact that surgical patients tended to have less co-morbidity and more of an isolated problem (Additional file 12).

The most frequently used trigger criterion was peripheral capillary oxygen saturation (SpO2) < 90% (Additional file 13). Interestingly, the incidence of SpO2 < 90% decreased by almost 10 per cent between MET activation and arrival (Additional file 8), which is believed to depend on the independent administration of oxygen by ward nurses and possibly also oxygen treatment recommendations over the phone pending the arrival of the MET. Despite this initial sign of improved optimisation, hypoxia and tachypnoea were associated with higher 30-day mortality among vital parameters, which strengthens the findings in previous studies [14]. Curiously, circulatory parameters did not play as important a role in predicting outcome in the MET patient population.

Caution should be taken concerning the fact that one of the vital parameters of the utmost importance in predicting 30-day mortality, i.e. the respiratory rate, was the parameter most often missing in the MET protocol – missing in more than every fifth patient. Respiratory abnormalities, such as tachypnoea and dyspnea, are well-recognised early warning signs in critically ill patients at risk of clinical deterioration and cardiac arrest [15, 16]. Considering the significant mortality risk when a patient presents with an elevated respiratory rate, we call for more attention to be paid to monitoring this vital parameter.

Despite hypoglycaemia being the most rarely encountered abnormal biomarker, it was associated with the highest mortality risk of all measured risk factors. Hypoglycaemia has previously been shown in several studies to be an independent risk factor for death in patients with acute illness [17–19]. Hence, it appears that disturbances in glucose metabolism signify an increased risk of adverse outcomes among critically ill patients. Other biomarkers associated with poor outcomes were hyperlactataemia, hypoxaemia and anaemia (Table 1). All the mortality-indicative biomarkers are found in regular blood gas analyses. Our data, however, revealed that arterial blood gases were missing during the care event for 40% of the patients. Routine arterial blood gas sampling was not included in the protocol. Instead, arterial blood gas sampling was performed depending on the clinician's assessment of the individual patient's clinical status. These variable circumstances lead us to speculate about whether more frequent blood gas sampling in clinically compromised patients would be beneficial for the early detection of severe illness and, by extension, improved outcome.

### Risk score for mortality

Previous prognostic risk scores within the MET field have only been presented to a limited extent, principally under dissimilar conditions or with differing endpoints, such as ICU admission and cardiac arrest [20, 21]. However, in parallel, a comparable study has been conducted in Australia, which supports our findings in a Scandinavian setting [22]. Similarly, a risk score was developed from the predictors in the multivariable regression model, with acceptable performance in estimating the probability of death following MET assessment. Thus, the concept of a risk score protocol could be a successful way to assist clinicians in identifying critically ill patients, optimising their care and ultimately reducing mortality.

The developed risk score showed a median AUC of 0.768 when corrected for optimism and the goodness of fit test indicated good calibration. Still, using the median risk score (= 14 points) as cut-off, specificity and PPV were rather low (less than two-thirds and less than half, respectively), indicating there is room for improvement. Even though the scoring system successfully identifies predictors, its accuracy still needs to be externally validated before it can be generally recommended in clinical practice. Subsequently, a refinement of the decision support can be achieved by monitoring trends of inpatients, with the use of artificial intelligence and machine learning techniques.

### Clinical implications

When patients are assessed by a MET team and triaged to the optimal level of care, several clinical factors are taken into account as decision support. The clinicians’ approach to further treatment will most likely differ, depending on experience and expertise. To achieve a more coherent handling of each patient case based on their condition, a risk scoring system, as described in this article, could be a useful tool in the decision-making process. The purpose of a risk score would be to serve as support for the clinician in prognosis prediction and mortality risk assessment, as a basis for the decision on treatment measures and escalation of the level of care. The purpose of a risk score is not to be a sole decision tool, but rather an additional part of all factors taken into account in the final decision-making. A large number of patient-related factors will contribute important information in the creation of a reliable risk score, including; age, type of ward for admittance, previous medical history, acute medical conditions, laboratory biomarkers, vital parameters and other clinical findings. The development of a risk score tool is still in the initial phase, thus, it needs to be emphasised that the risk score described in this article needs further testing and validation on larger sample sizes before final implementation in clinical practice. Also, with the help of artificial intelligence and machine learning techniques, it is probably possible to further improve the accuracy of the model [23–25]. In addition, hospital-specific risk scores may need to be developed for logistical reasons, such as varying patient cohorts in the wards. With these considerations in mind, the availability of a standardised risk score for the estimation of the mortality risk at MET assessment should be favourable from a prioritisation and optimisation perspective.

### Study strengths and limitations

#### Strengths

The study was population-based with well-defined inclusion and exclusion criteria and a relatively large sample size. Furthermore, it was consecutive, and all cases were evaluated for inclusion. Moreover, it was chart based, with all cases handled manually.

#### Limitations

Data were limited to 2010–2015, and new conditions may have emerged since then. The 'MET dose', calculated as the number of MET assessments divided by the number of hospital admissions (approximately 12/1,000), was low in comparison to studies in other healthcare systems, possibly indicating an inefficiency in the system [26]. Despite this, our 'MET dose' is higher than the dose previously reported in a before-and-after trial in Sweden, where the implementation of MET was associated with a significant improvement in cardiac arrest rate and in-hospital mortality [27]. Given the retrospective design, it was not possible to check and correct for afferent limb failure, i.e. delayed activation of the MET [28]. Further, it was a single-centre study using a single-parameter system, not fully transferable to hospitals with different routines. Since it was a retrospective study, we were not able to control for unmeasured factors. Also, for several of the variables, the number of missing data was substantial, although we tried to handle this by using multiple imputation methods in the multivariable analysis. In a retrospective register study, it is not possible to draw any conclusion regarding the cause and the effect. We are only able to describe associations.

## Conclusions

The MET population remains an exposed category amongst hospitalised patients. In a cohort of 2,601 patients assessed by the MET, the overall 30-day mortality was 29.0%. We identified thirteen factors independently associated with 30-day mortality concerning; age, type of ward for admittance, vital parameters, laboratory biomarkers, previous medical history and acute medical condition. A prognostic risk score was developed, based on these independent factors available at the time of MET assessment. The MET risk score was shown to successfully identify patients at high risk of dying within 30 days. The risk score could thus serve as a complementary tool in the early identification of prognostically poor patients and decision-making for further treatment efforts and escalation of the level of care. However, it needs to be emphasised that further refinement of the risk score would be desirable in regard to specificity. In addition, an external validation needs to be performed before final implementation.

## Supplementary Information

**Additional file 1. **

**Additional file 2. **

**Additional file 3. **

**Additional file 4. **

**Additional file 5. **

**Additional file 6. **

**Additional file 7. **

**Additional file 8. **

**Additional file 9. **

**Additional file 10. **

**Additional file 11. **

**Additional file 12. **

**Additional file 13. **

## Data Availability

The datasets used and analysed during the current study are available from the corresponding author upon reasonable request.

## Abbreviations

MET: Medical emergency teams
ICU: Intensive care unit
CAT: Cardiac arrest team
LOMT: Limitations of medical therapy
