# Supplementary material for: Outcome prediction for patients assessed by the medical emergency team: a retrospective cohort study
Source: BMC Emerg Med. 2022 Dec 9;22:200. doi: 10.1186/s12873-022-00739-w (PMC9733206; doi:10.1186/s12873-022-00739-w)
Supplement: Supplementary file 1 — Additional file 1. [file 12873_2022_739_MOESM1_ESM.pdf]

Additional file 1

a) MEDICAL EMERGENCY TEAM (MET) DOSE

The number of MET activations per 1,000 admissions.

YEAR	REGISTER SAMPLE (3,553)	STUDY SAMPLE (2,601)	HOSPITAL ADMISSIONS
2010	573 (11.5)	437 (8.8)	49,834
2011	609 (12.3)	428 (8.6)	49,678
2012	601 (12.2)	449 (9.2)	49,071
2013	543 (11.0)	406 (8.2)	49,356
2014	567 (11.8)	407 (8.5)	47,837
2015	660 (14.3)	474 (10.3)	46,071

Additional file 1a. *The annual number of MET assessments (columns 2 and 3) and hospital admissions (column 4), including MET dose within the respective brackets*

b) ANNUAL DISTRIBUTION OF ICU ADMISSIONS AND OUTCOME

YEAR	ICU ADMISSION (%)	DEATH AT WARD (%)	DEATH AT ICU (%)
2010	44.2	27.8	31.4
2011	51.1	32.2	24.8
2012	44.9	30.8	28.3
2013	42.2	29.2	27.3
2014	42.4	26.5	28.7
2015	41.3	31.7	33.2

Additional file 1b. *The annual percentage of ICU admission (column 2), death within 30 days at general wards (column 3) and ICU (column 4), following MET assessment*
