# Supplementary material for: Outcome prediction for patients assessed by the medical emergency team: a retrospective cohort study
Source: BMC Emerg Med. 2022 Dec 9;22:200. doi: 10.1186/s12873-022-00739-w (PMC9733206; doi:10.1186/s12873-022-00739-w)
Supplement: Supplementary file 2 — Additional file 2. [file 12873_2022_739_MOESM2_ESM.pdf]

Additional file 2

When comparing patients requiring one MET assessment to those requiring one or more additional assessments during the same hospitalisation period, there were no significant differences in gender balance or 30-day mortality. In terms of previous medical history, patients with additional MET assessments significantly more often presented haematological diseases, whereas those with only one MET assessment were significantly older.

PATIENTS WITH ADDITIONAL MET ASSESSMENTS

**AGE, GENDER, PREVIOUS MEDICAL HISTORY* AND OUTCOME**	**No (n=2,061)**	**Yes (n=540)**	**p#**
AGE; years (mean/median)	66.1/69	64.0/67	0.003
FEMALE GENDER	45.1	41.1	0.11
PREVIOUS MEDICAL HISTORY:			
Myocardial infarction	9.7	10.6	0.25
Angina pectoris	13.7	11.1	0.30
Cardiac failure	12.0	8.1	0.09
Other cardiac diseases**	21.3	20.4	0.44
Cardiac arrest	0.9	0.4	0.24
Stroke	9.7	9.1	0.99
Hypertension	31.3	33.5	0.07
Peripheral arterial disease	4.2	3.5	0.64
Pulmonary disease	24.2	22.0	0.38
Respiratory insufficiency	4.9	3.5	0.22
Gastrointestinal disease	13.4	14.8	0.54
Liver disease	10.1	8.9	0.16
Pancreatic disease	2.7	1.9	0.20
Renal disease	10.3	8.9	0.29
Endocrine disease	1.8	1.7	0.82
Haematological disease	6.1	9.8	0.006
Cancer	30.8	26.5	0.04
Skeletal disease	11.7	8.7	0.09
Rheumatic disease	6.8	8.7	0.13
Diabetes	16.6	15.4	0.43
Neurological disease	17.9	22.0	0.02
Psychiatric disease	4.5	3.9	0.38
Addiction	9.7	8.9	0.25
30-DAY MORTALITY	30.0	25.4	0.18

**Information on previous medical history was missing for one patient*

*** Including cardiac arrhythmias, valvular heart diseases, pericardial disorders, cardiogenetic disorders or congenital heart defects, among others*

# Age-adjusted (except for age itself) p-value by multivariable logistic regression, for difference between the two groups.

Additional file 2. *Baseline characteristics and outcome in relation to whether patients required additional MET assessment or not while hospitalised in 2010-2015 at Sahlgrenska University Hospital*
