# Supplementary material for: Outcome prediction for patients assessed by the medical emergency team: a retrospective cohort study
Source: BMC Emerg Med. 2022 Dec 9;22:200. doi: 10.1186/s12873-022-00739-w (PMC9733206; doi:10.1186/s12873-022-00739-w)
Supplement: Supplementary file 3 — Additional file 3. [file 12873_2022_739_MOESM3_ESM.pdf]

3. Data collection form

Year (2010-2015) □□□□

Serial MET number □□□□

Personal number □□ □□ □□-□□□□

Gender (M/F) □

Date of hospital admittance □□□□ □□ □□

Date of MET assessment □□□□ □□ □□

MET call number (for same hospital episode) □□

Type of ward □□

01 = Geriatric
02 = Hand surgical
03 = Cardiology
04 = Dermatology
05 = Surgical
06 = Gynaecological
07 = Respiratory
08 = General medical
09 = Oncology
10 = Orthopaedics
11 = Plastic surgical
12 = Psychiatric
13 = Rheumatology
14 = Urological
15 = Ear, nose and throat
16 = Neurological (including spinal injury and neuro surgical wards bleeding)
17 = Vascular surgical
18 = Transplantation

Previous medical history Yes/No

Condition	Yes/No
Angina pectoris	☐
Myocardial infarction	☐
Cardiac failure	☐
Cardiac arrest	☐
Pulmonary disease	☐
Respiratory insufficiency	☐
Hypertension	☐
Diabetes	☐
Renal disease	☐
Stroke	☐
Neurological disease	☐
Periopheral arterial disease (claudicatio, carotis stenosis, etc)	☐
Rheumatic disease	☐
Liver disease	☐
Pancreatic disease	☐
Gastrointestinal disease	☐
Cancer	☐
Psychiatric disease	☐
Endocrine disease	☐
Addiction	☐
Other cardiac diseases (valvular disorders, arrhythmia, etc)	☐
Haematological disease	☐

Skeletal disease ☐

Other disease ☐

If yes, specify; ……………………………………………………………………..

Trigger criteria

	Yes/No
Threatened airway	☐
Respiratory rate < 8 or > 30 breaths/min	☐
POX < 90%	☐
Heart rate < 40 or > 130 beats/min	☐
Systolic blood pressure < 90 mm Hg	☐
Decreased consciousness	☐
Serious concern	☐

Status at arrival

Respiratory rate	☐☐ breaths/min
Heart rate	☐☐☐ beats/min
Consciousness (RLS 1-8)	☐
POX	☐☐☐ %
Oxygen (Yes = 1, No = 0)	☐
Systolic blood pressure	☐☐☐ mmHg

Measures at the ward Yes/No
(already taken or prescribed)

Oxygen ☐

Intravenous fluid ☐

Medical treatment ☐
If yes, specify; …………………………………………………………………..

Blood transfusion ☐

Laboratory test ☐

Other ☐
If yes, specify; …………………………………………………………………..

Level of care ☐

1. No treatment limitation
2. Treatment limitations
   If yes, specify; …………………………………………………………………..
3. Palliation decision

LOMT or DNAR decision Yes/No
(resulting from MET assessment)

☐

MET assessment ☐

1. No measures / No medical indication
2. No intensive care due to LOMT-decision
3. Stabilisation at nursing ward
4. Transferral to ICU
5. Transferral to specialised ward / specialised ICU / another hospital
6. Delayed transferral to ICU

Laboratory findings

(# if not available)

pH	□,□□pH
pCO2	□□,□kPa
pO2	□□,□kPa
Base excess	□□□,□+/-
Saturation O2	□□□%
Haemoglobin	□□□g/l
Sodium	□□□mmol/l
Potassium	□,□ mmol/l
Calcium (Ionised) □	□,□□mmol/l
Glukos	□□,□ mmol/l
Serum lactate	□□,□mmol/l
Haematocrit	□□□l/l
Creatinine	□□□□µmol/l

Follow-up: Serum lactate in ICU □□,□mmol/l

Type of blood gas: □

1. Arterial
2. Venous
0. No blood gas

Location for first blood gas: □

1. Regular ward
2. ICU

Acute medical condition at MET assessment Yes/No

Acute coronary syndrome ☐

Sepsis ☐

Pneumonia ☐

Gastroenteritis ☐

Postoperative infection ☐

Other infection ☐

Other postoperative complications ☐

Pulmonary embolism ☐

Cardiac failure ☐

Pulmonary disease ☐

Renal failure ☐

Clinically relevant haemorrhage ☐

Allergic reaction/anaphylaxis ☐

Other ☐

If yes, specify; …………………………………………………………………..

Result after MET assessment Yes/No

Cardiac arrest (where CPR is initiated) ☐

If yes; Date ☐☐☐☐ ☐☐ ☐☐

Mortality < 30 days ☐

If yes; Date ☐☐☐☐ ☐☐ ☐☐

Mortality < 1 year ☐

If yes; Date ☐☐☐☐ ☐☐ ☐☐

Mortality location: ☐

1. ICU
2. Regular ward
3. Other

Date of ICU admittance ☐☐☐☐ ☐☐ ☐☐

Date of ICU discharge ☐☐☐☐ ☐☐ ☐☐

Date of hospital discharge ☐☐☐☐ ☐☐ ☐☐

Primary diagnosis (ICD 10) ☐☐☐☐☐

(for the care event)

Primary diagnosis in writing……………………………………………………………………………………………
