# Supplementary material for: Outcome prediction for patients assessed by the medical emergency team: a retrospective cohort study
Source: BMC Emerg Med. 2022 Dec 9;22:200. doi: 10.1186/s12873-022-00739-w (PMC9733206; doi:10.1186/s12873-022-00739-w)
Supplement: Supplementary file 4 — Additional file 4. [file 12873_2022_739_MOESM4_ESM.pdf]

Additional file 4

Most of the patients assessed by the MET were admitted to general medicine wards, followed by general surgery and neurological wards. Higher 30-day mortality was found in geriatric wards, followed by respiratory medicine and oncology wards. Overall, there was a similar number of patients from any medical and surgical wards. MET assessed patients on surgical wards were, however, associated with a significantly lower age-adjusted mortality during the subsequent 30 days.

TYPE OF WARD

**TYPE OF WARD**	**DEATH WITHIN 30 DAYS** Yes (n=755)	No (n=1,846)	**Age adjusted OR (95% CI)**	**p#**
MEDICAL WARDS	423 (56.0)	718 (38.9)	2.19 (1.83,2.62)	<0.0001
General medicine	234 (31.0)	451 (24.4)		
Oncology	68 (9.0)	99 (5.4)		
Respiratory medicine	61 (8.1)	66 (3.6)		
Cardiology	31 (4.1)	60 (3.3)		
Rheumatology	15 (2.0)	29 (1.6)		
Geriatric	13 (1.7)	10 (0.5)		
Dermatology	1 (0.1)	3 (0.2)		
SURGICAL WARDS	230 (30.5)	929 (50.3)	0.38 (0.31,0.46)	<0.0001
General surgery	131 (17.4)	403 (21.8)		
Urology	27 (3.6)	194 (10.5)		
Transplantation	21 (2.8)	107 (5.8)		
Orthopaedics	11 (1.5)	64 (3.5)		
Gynaecology	14 (1.9)	54 (2.9)		
Vascular surgery	18 (2.4)	51 (2.8)		
Plastic surgery	2 (0.3)	25 (1.4)		
Ear, nose and throat	6 (0.8)	25 (1.4)		
Hand surgery	0 (0.0)	6 (0.3)		
NEUROLOGICAL WARDS*	101 (13.4)	183 (9.9)	1.48 (1.13,1.94)	0.004
PSYCHIATRIC WARDS	1 (0.1)	16 (0.9)	0.26 (0.03,2.01)	0.28##

Results presented as number (per cent)

** Neurological wards include neurology, spinal injury and neurosurgery patients*

# Age-adjusted p-value for association with 30-day mortality

Firth bias correction used for likelihood penalty

OR, odds ratio; CI, confidence interval

Additional file 4. *Outcome in relation to the type of ward for patients where MET was activated while hospitalised in 2010-2015 at Sahlgrenska University Hospital*
