# Supplementary material for: Outcome prediction for patients assessed by the medical emergency team: a retrospective cohort study
Source: BMC Emerg Med. 2022 Dec 9;22:200. doi: 10.1186/s12873-022-00739-w (PMC9733206; doi:10.1186/s12873-022-00739-w)
Supplement: Supplementary file 5 — Additional file 5. [file 12873_2022_739_MOESM5_ESM.pdf]

Additional file 5

The most frequently reported conditions in the patients' previous medical history were cancer, hypertension, and pulmonary disease. Previous conditions associated with higher 30-day mortality were cardiac failure, followed by haematological disease, angina pectoris and pulmonary disease. The following previous conditions were associated with a significantly higher age-adjusted mortality during the subsequent 30 days: cancer, haematological disease, pulmonary disease, and liver disease.

PREVIOUS MEDICAL HISTORY

PREVIOUS MEDICAL HISTORY*	DEATH WITHIN 30 DAYS Yes (n=755)	No (n=1,846)	Age adjusted OR (95% CI)	p#
Cancer	276 (36.6)	502 (27.2)	1.38 (1.14,1.66)	0.0007
Hypertension	264 (35.0)	562 (30.4)	0.83 (0.69,1.01)	0.06
Angina pectoris	131 (17.4)	211 (11.4)	1.13 (0.88,1.44)	0.34
Myocardial infarction	93 (12.3)	163 (8.8)	1.04 (0.78,1.37)	0.80
Cardiac failure	120 (15.9)	171 (9.3)	1.31 (1.01,1.70)	0.04
Cardiac arrest	6 (0.8)	15 (0.8)	0.71 (0.27,1.85)	0.48
Other cardiac diseases**	199 (26.4)	349 (18.9)	0.99 (0.80,1.23)	0.95
Peripheral arterial disease	30 (4.0)	75 (4.1)	0.70 (0.45,1.08)	0.11
Stroke	82 (10.9)	167 (9.0)	0.88 (0.66,1.17)	0.36
Neurological disease	146 (19.4)	342 (18.5)	1.01 (0.80,1.26)	0.96
Haematological disease	69 (9.2)	110 (6.0)	1.98 (1.42,2.76)	<0.0001
Diabetes	128 (17.0)	297 (16.1)	0.97 (0.76,1.22)	0.77
Endocrine disease	11 (1.5)	36 (2.0)	0.71 (0.35,1.44)	0.35
Rheumatic disease	53 (7.0)	134 (7.3)	0.91 (0.65,1.28)	0.60
Pulmonary disease	228 (30.2)	389 (21.1)	1.38 (1.14,1.68)	0.001
Respiratory insufficiency	39 (5.2)	80 (4.3)	1.01 (0.67,1.50)	0.98
Gastrointestinal disease	95 (12.6)	262 (14.2)	0.89 (0.68,1.15)	0.36
Liver disease	81 (10.7)	175 (9.5)	1.61 (1.20,2.16)	0.001
Pancreatic disease	20 (2.7)	46 (2.5)	1.16 (0.67,2.00)	0.60
Renal disease	78 (10.3)	183 (9.9)	1.04 (0.78,1.39)	0.79

Skeletal disease	100 (13.3)	187 (10.1)	1.16 (0.89,1.52)	0.27
Psychiatric disease	18 (2.4)	96 (5.2)	0.58 (0.34,0.97)	0.04
Addiction	56 (7.4)	192 (10.4)	1.00 (0.72,1.38)	1.00

Results presented as number (per cent)

** Information on previous medical history was missing for one patient who died within 30 days*

*** Including; cardiac arrhythmias, valvular heart diseases, pericardial disorders, cardiogenetic disorders or congenital heart defects, among others*

# Age-adjusted p-value for association with 30-day mortality

OR, odds ratio; CI, confidence interval

Additional file 5. *The outcome in relation to previous medical history for patients where MET was activated while hospitalised in 2010-2015 at Sahlgrenska University Hospital*
