# Supplementary material for: Outcome prediction for patients assessed by the medical emergency team: a retrospective cohort study
Source: BMC Emerg Med. 2022 Dec 9;22:200. doi: 10.1186/s12873-022-00739-w (PMC9733206; doi:10.1186/s12873-022-00739-w)
Supplement: Supplementary file 6 — Additional file 6. [file 12873_2022_739_MOESM6_ESM.pdf]

Additional file 6

The acute conditions most frequently associated with MET activation were sepsis and pneumonia. Acute conditions associated with higher 30-day mortality were gastroenteritis, acute coronary syndrome, cardiac failure and renal failure. The following acute medical conditions were associated with a significantly higher age-adjusted mortality during the subsequent 30 days: cardiac failure, pneumonia and renal failure. Other infections (exemplified in the footnote), postoperative infection and other postoperative complications (exemplified in the footnote) were associated with a significantly lower mortality.

ACUTE MEDICAL CONDITION

ACUTE MEDICAL CONDITION*	DEATH WITHIN 30 DAYS Yes (n=755)	No (n=1,846)	Age adjusted OR (95% CI)	p#
Acute coronary syndrome	43 (5.7)	57 (3.1)	1.46 (0.96,2.21)	0.07
Cardiac failure	137 (18.2)	196 (10.8)	1.47 (1.15,1.88)	0.002
Pulmonary disease	73 (9.7)	110 (6.1)	1.44 (1.04,1.97)	0.03
Pulmonary embolism	35 (4.6)	75 (4.1)	1.10 (0.72,1.67)	0.67
Pneumonia	232 (30.8)	375 (20.7)	1.63 (1.33,1.99)	<0.0001
Sepsis	254 (33.7)	592 (32.7)	1.10 (0.91,1.32)	0.32
Other infection**	110 (14.6)	454 (25.0)	0.50 (0.39,0.63)	<0.0001
Gastroenteritis	10 (1.3)	10 (0.6)	2.13 (0.86,5.27)	0.10
Renal failure	82 (10.9)	121 (6.7)	1.75 (1.29,2.38)	0.0003
Clinically relevant haemorrhage	103 (13.7)	258 (14.2)	0.88 (0.68,1.13)	0.32
Postoperative infection	33 (4.4)	176 (9.7)	0.44 (0.30,0.66)	<0.0001
Other postoperative complications***	41 (5.4)	206 (11.4)	0.44 (0.31,0.63)	<0.0001
Allergic reaction/anaphylaxis	0 (0.0)	34 (1.9)	0.05 (0.00,0.84)##	0.04##

Results presented as number (per cent)

** Information on the acute medical condition was missing for 1 and 33 patients in the two groups, respectively*

*** Including pancreatitis, cholecystitis, pyelonephritis, diverticulitis, peritonitis, pancytopenia, neutropenic fever, cerebral abscess or meningitis, among others*

**** Including respiratory insufficiency, pneumothorax, deep vein thrombosis, postoperative cerebral insult, hypovolemia, intestinal perforation or anastomotic leakage, among others*

# Age-adjusted p-value for association with 30-day mortality

## Firth bias correction used for likelihood penalty

OR, odds ratio; CI, confidence interval

Additional file 6. *The outcome in relation to acute medical condition for patients where MET was activated while hospitalised in 2010-2015 at Sahlgrenska University Hospital*
