# Supplementary material for: Outcome prediction for patients assessed by the medical emergency team: a retrospective cohort study
Source: BMC Emerg Med. 2022 Dec 9;22:200. doi: 10.1186/s12873-022-00739-w (PMC9733206; doi:10.1186/s12873-022-00739-w)
Supplement: Supplementary file 7 — Additional file 7. [file 12873_2022_739_MOESM7_ESM.pdf]

Additional file 7

The most frequent biomarker alteration was hyperglycaemia, followed by low haemoglobin, hypoxaemia and elevated serum creatinine. Biomarkers associated with higher 30-day mortality were acidosis, hypoxaemia, hyponatraemia, hypernatraemia, hyperkalaemia, hypoglycaemia, elevated serum creatinine and hyperlactataemia. The same biomarkers were also associated with a significantly higher age-adjusted mortality during the subsequent 30 days, with the exception of elevated serum creatinine and the addition of hypocapnia, hypercapnia, and low haemoglobin.

LABORATORY BIOMARKERS

LABORATORY BIOMARKERS	DEATH WITHIN 30 DAYS Yes (n=755)	No (n=1,846)	p#
pH (224/597)*			
<7.25	89 (16.8)	90 (7.2)	<0.0001
pCO2; kPa (221/591)			
<4.0	84 (15.7)	143 (11.4)	0.004
>8.0	89 (16.7)	131 (10.4)	0.003
pO2; kPa (275/753)			
median (10th,90th percentile)	9.1 (5.5,16.8)	10.0 (5.9,18,9)	0.002
Haemoglobin (Hb); g/l (94/169)			
<90	132 (20.0)	275 (16.4)	0.001
>150	45 (6.8)	79 (4.7)	0.02
Sodium (Na); mmol/l (73/173)			
<130	77 (11.3)	131 (7.8)	0.002
>145	52 (7.6)	66 (3.9)	0.002
Potassium (K); mmol/l (78/178)			
<3.0	24 (3.5)	57 (3.4)	0.79
>5.5	49 (7.2)	42 (2.5)	<0.0001
Calcium (Ca); mmol/l (281/682)			
median (10th,90th percentile)	1.17 (1.06,1.29)	1.16 (1.06,1.26))	0.79
Glucose; mmol/l (282/673)			
<4.2	23 (4.9)	18 (1.5)	<0.0001
>10.0	15 (32.8)	297 (25.3)	0.03
Creatinine; µmol/l (80/188)			
median (10th,90th percentile)	112 (52,280)	90 (50,240)	0.01
Lactate; mmol/l (288/701)			
median (10th,90th percentile)	2.1 (1.0,8.4)	1.6 (0.8,4.2)	<0.0001

Results presented as number (per cent)

** Number of patients for whom information was missing in the two groups, respectively*

# Age-adjusted p-value for association with 30-day mortality

Additional file 7. *The outcome in relation to laboratory biomarkers for patients where MET was activated while hospitalised in 2010-2015 at Sahlgrenska University Hospital*
