# Supplementary material for: Outcome prediction for patients assessed by the medical emergency team: a retrospective cohort study
Source: BMC Emerg Med. 2022 Dec 9;22:200. doi: 10.1186/s12873-022-00739-w (PMC9733206; doi:10.1186/s12873-022-00739-w)
Supplement: Supplementary file 8 — Additional file 8. [file 12873_2022_739_MOESM8_ESM.pdf]

Additional file 8

The most frequent abnormalities in terms of vital parameters were hypoxia and tachypnoea. Patients who presented with these findings also had higher 30-day mortality. The following vital parameters were associated with a significantly higher age-adjusted mortality during the subsequent 30 days: hypoxia, tachypnoea, tachycardia and unconsciousness.

STATUS ON ARRIVAL OF MET

	**DEATH WITHIN 30 DAYS**		
**VITAL PARAMETERS**	**Yes (n=755)**	**No (n=1,846)**	**p#**
SpO2; % (15/41)*			<0.0001
<90	338 (45.7)	408 (22.6)	
90-95	273 (36.9)	684 (37.9)	
>95	129 (17.4)	713 (39.5)	
RR; breaths/min (128/436)			
median (10th,90th percentile)	28 (16,40)	24 (14,37)	<0.0001
HR; beats/min (24/36)			
median (10th,90th percentile)	104 (70,140)	100 (69,134)	<0.0001
SBP; mmHg (43/64)			
<90	164 (23.0)	336 (18.8)	0.06
Consciousness; RLS (160/260)			<0.0001
1	315 (52.9)	1103 (69.5)	
2-3	232 (39.0)	410 (25.9)	
>3	48 (8.1)	73 (4.6)	

Results presented as number (per cent)

** Number of patients for whom information was missing in the two groups, respectively*

# Age-adjusted p-value for association with 30-day mortality

SpO2, peripheral capillary oxygen saturation; RR, respiratory rate; HR, heart rate; SBP, systolic blood pressure; RLS, reaction level scale

Additional file 8. *The outcome in relation to status on arrival of MET for patients where MET was activated while hospitalised in 2010-2015 at Sahlgrenska University Hospital*
