# Supplementary material for: Outcome prediction for patients assessed by the medical emergency team: a retrospective cohort study
Source: BMC Emerg Med. 2022 Dec 9;22:200. doi: 10.1186/s12873-022-00739-w (PMC9733206; doi:10.1186/s12873-022-00739-w)
Supplement: Supplementary file 9 — Additional file 9. [file 12873_2022_739_MOESM9_ESM.pdf]

Additional file 9

a) AGE AND GENDER

Overall 30-day mortality was 29.0%. Higher age was associated with a significantly higher 30-day mortality. There were no significant differences in 30-day mortality with regard to gender.

	INCIDENCE n (%)	30-DAY MORTALITY (%)	OR (95% CI)	p
AGE; years		29.0	1.042 (1.035,1.049)	<0.0001
mean±sd	65.7±16.8			
median	68			
FEMALE GENDER	1,152 (44.3)	28.0	0.91 (0.77,1.08)	0.28
			0.92 (0.77,1.10)#	0.37#

# Age adjusted

OR, odds ratio; CI, confidence interval

Additional file 9a. *The outcome in relation to age and gender in the study sample (n=2,601)*

b) LEVEL OF TREATMENT AND LEVEL OF CARE

Patients with palliative decisions and treatment limitations demonstrated a significantly higher 30-day mortality, in comparison to patients without any treatment restrictions. There were no significant differences in 30-day mortality with regard to the level of care after MET assessment.

	LOMT (*n*=458)	NO LOMT (*n*=2,143)	p#	REGULAR WARD (*n*=1,386)	TRANSFERRED TO ICU (*n*=1,105)	p#
30-DAY MORTALITY	65.5%	21.2%	<0.0001	29.9%	28.9%	0.31

# Age-adjusted p-value

LOMT, limitation of medical therapy; ICU, intensive care unit

Additional file 9b. *The outcome in relation to the level of treatment and level of care in the study sample (n=2,601)*
