# Supplementary figures and images for: Outcome prediction for patients assessed by the medical emergency team: a retrospective cohort study
Source: BMC Emerg Med. 2022 Dec 9;22:200. doi: 10.1186/s12873-022-00739-w (PMC9733206; doi:10.1186/s12873-022-00739-w)

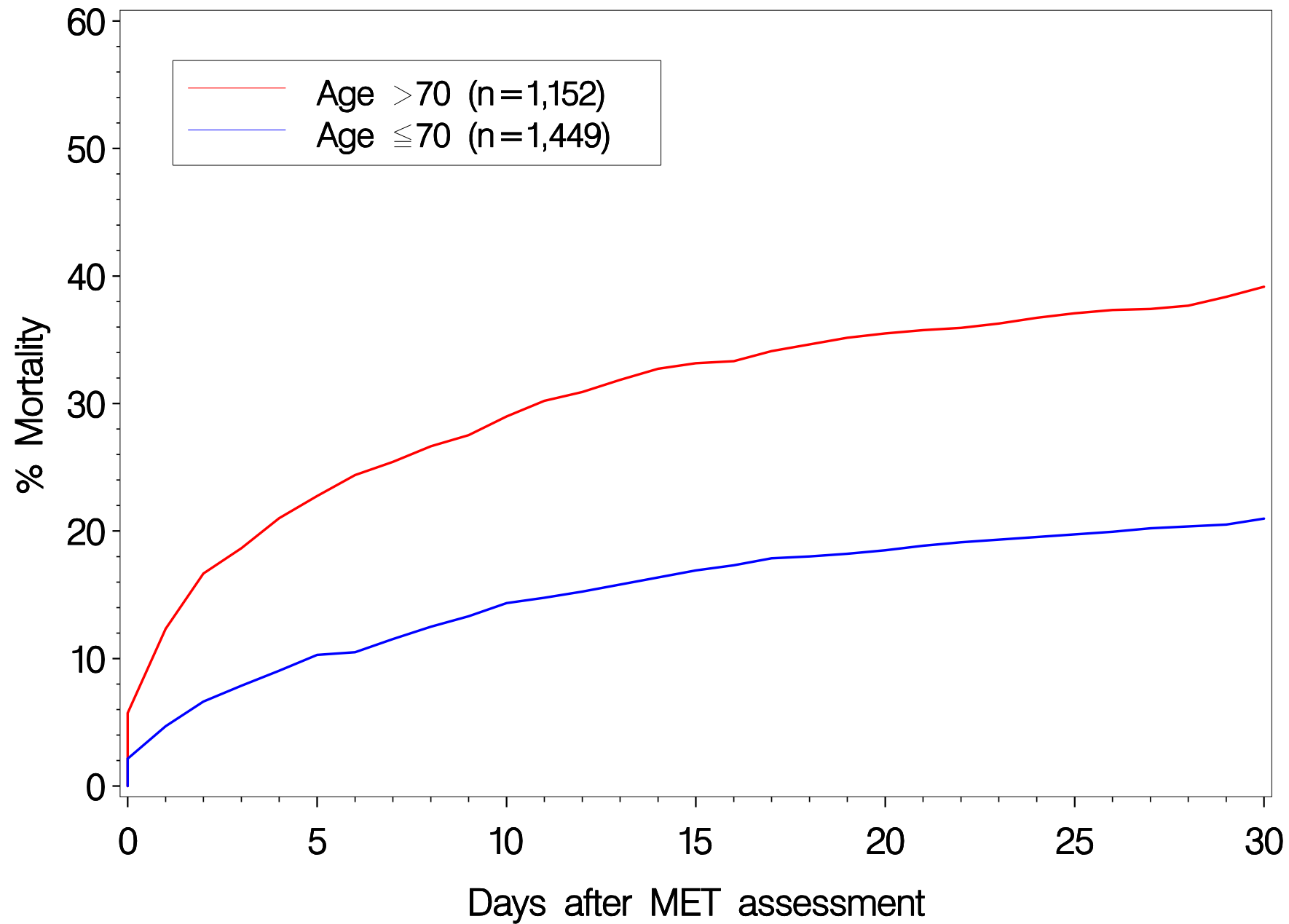

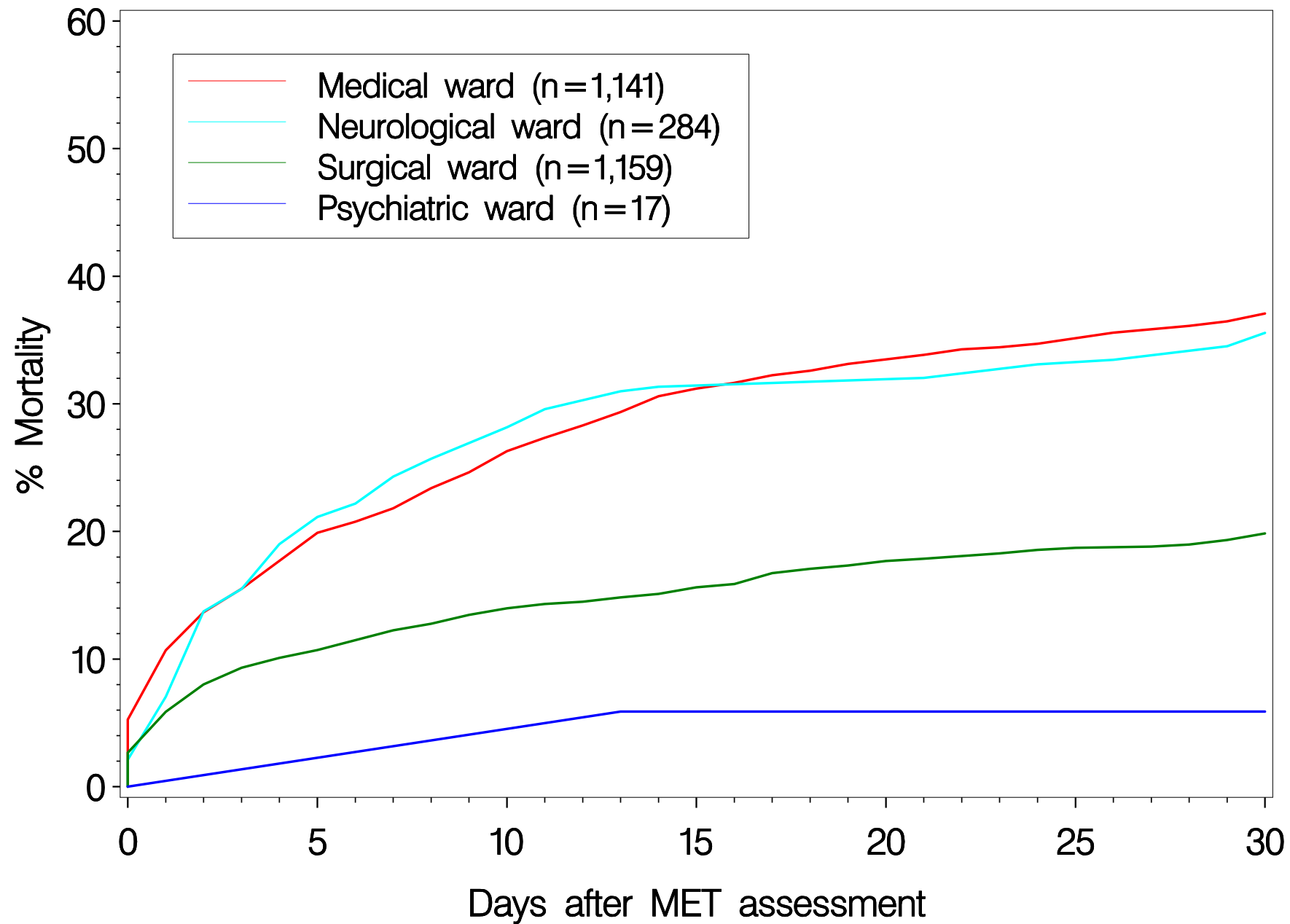

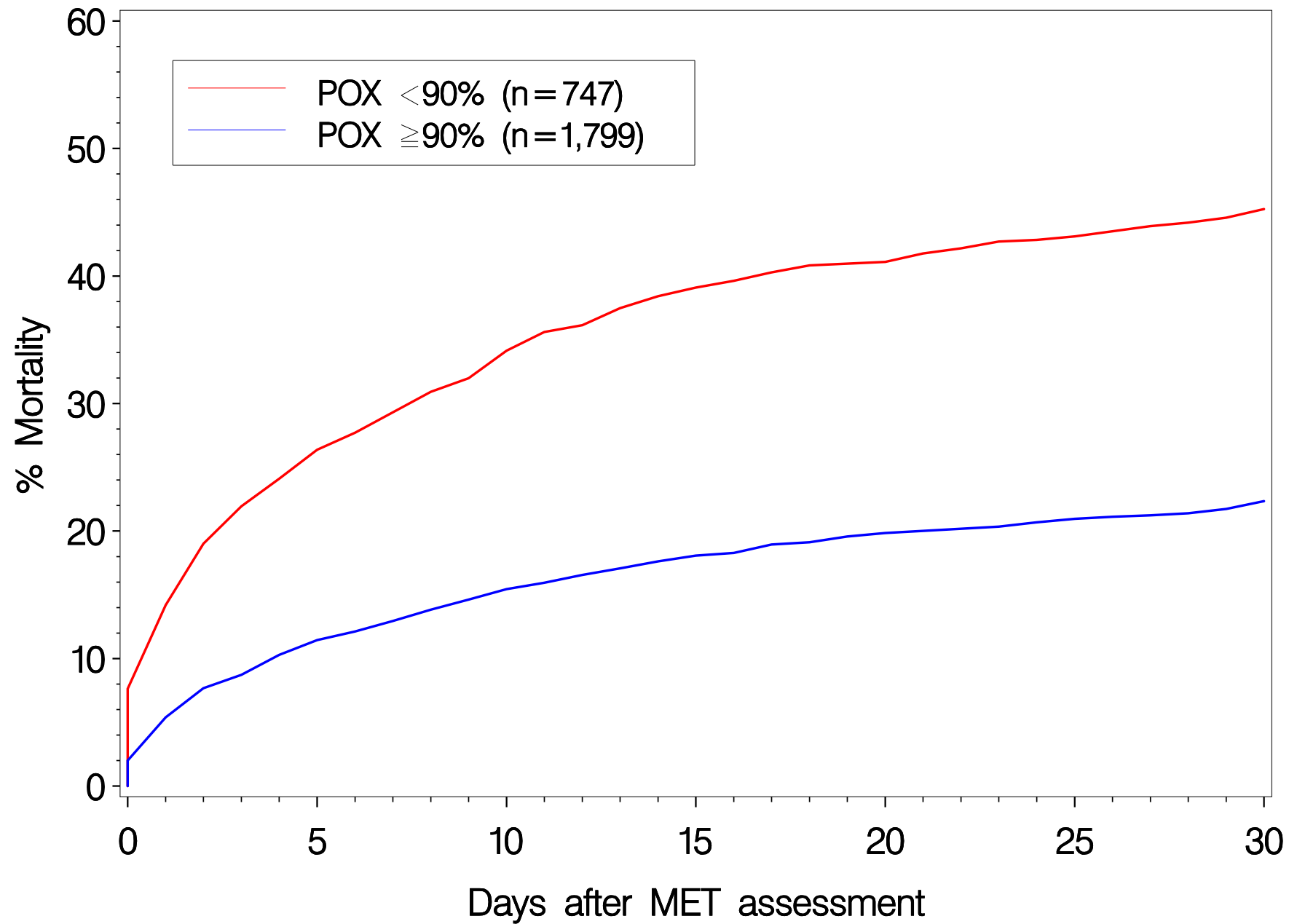

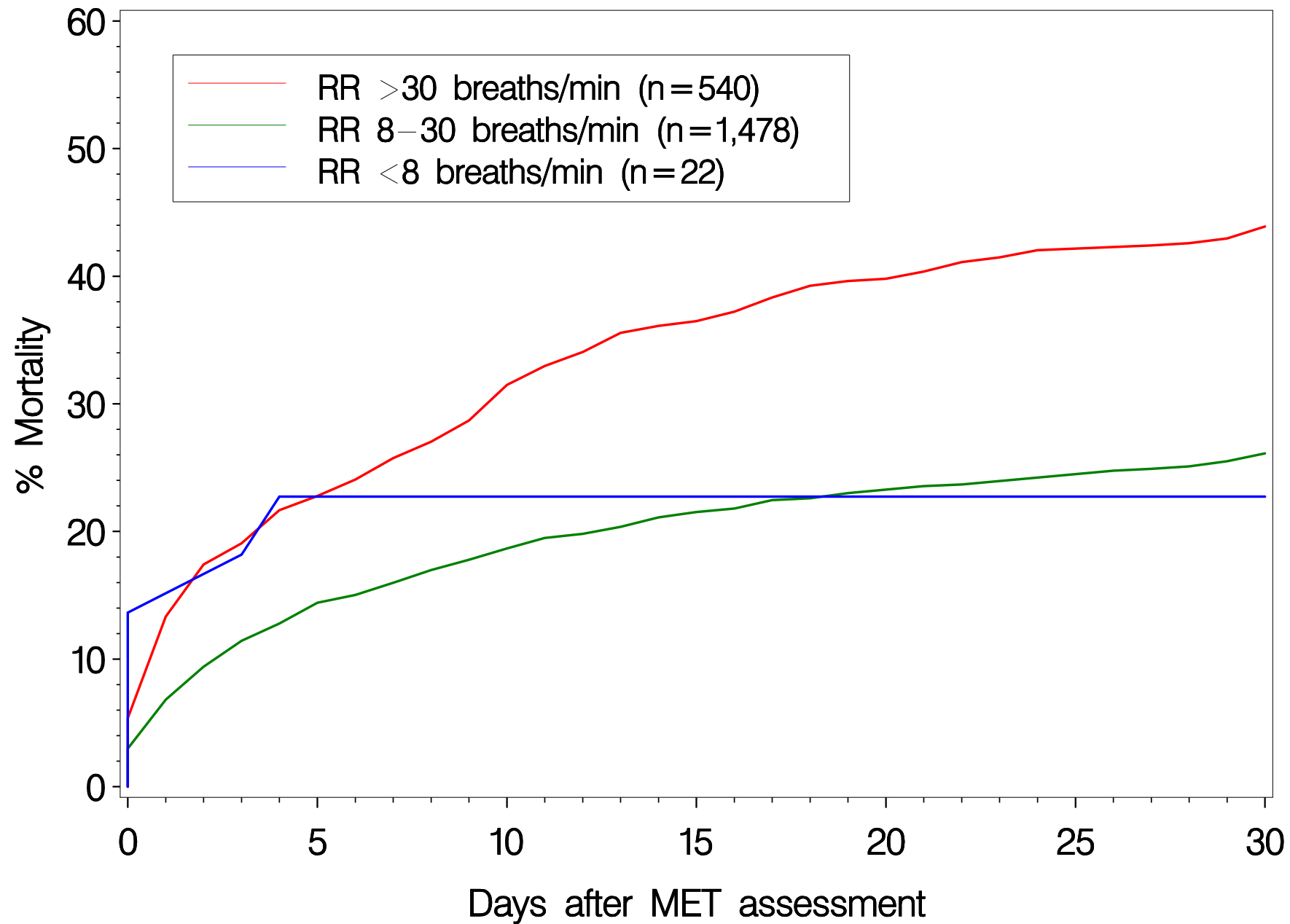

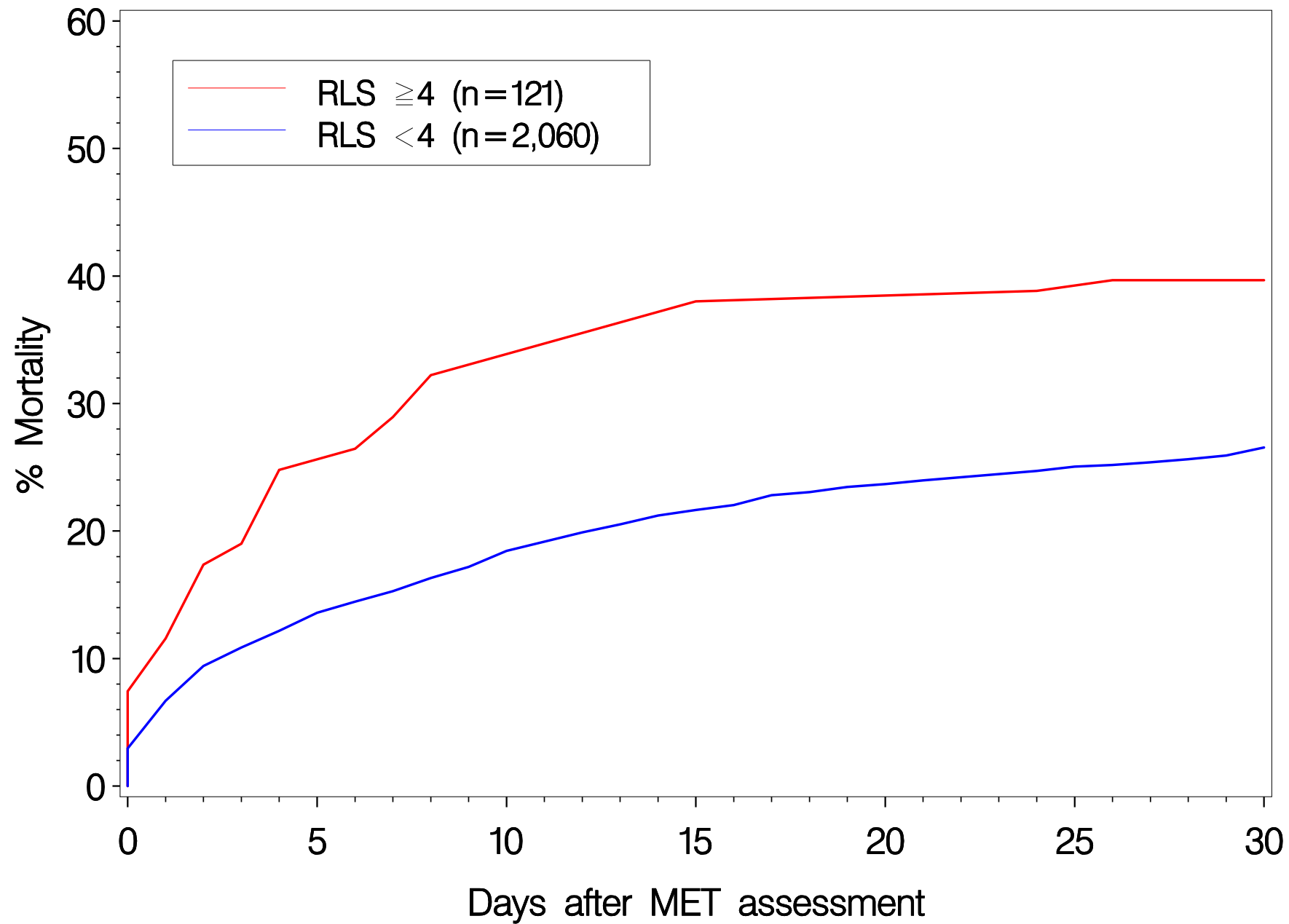

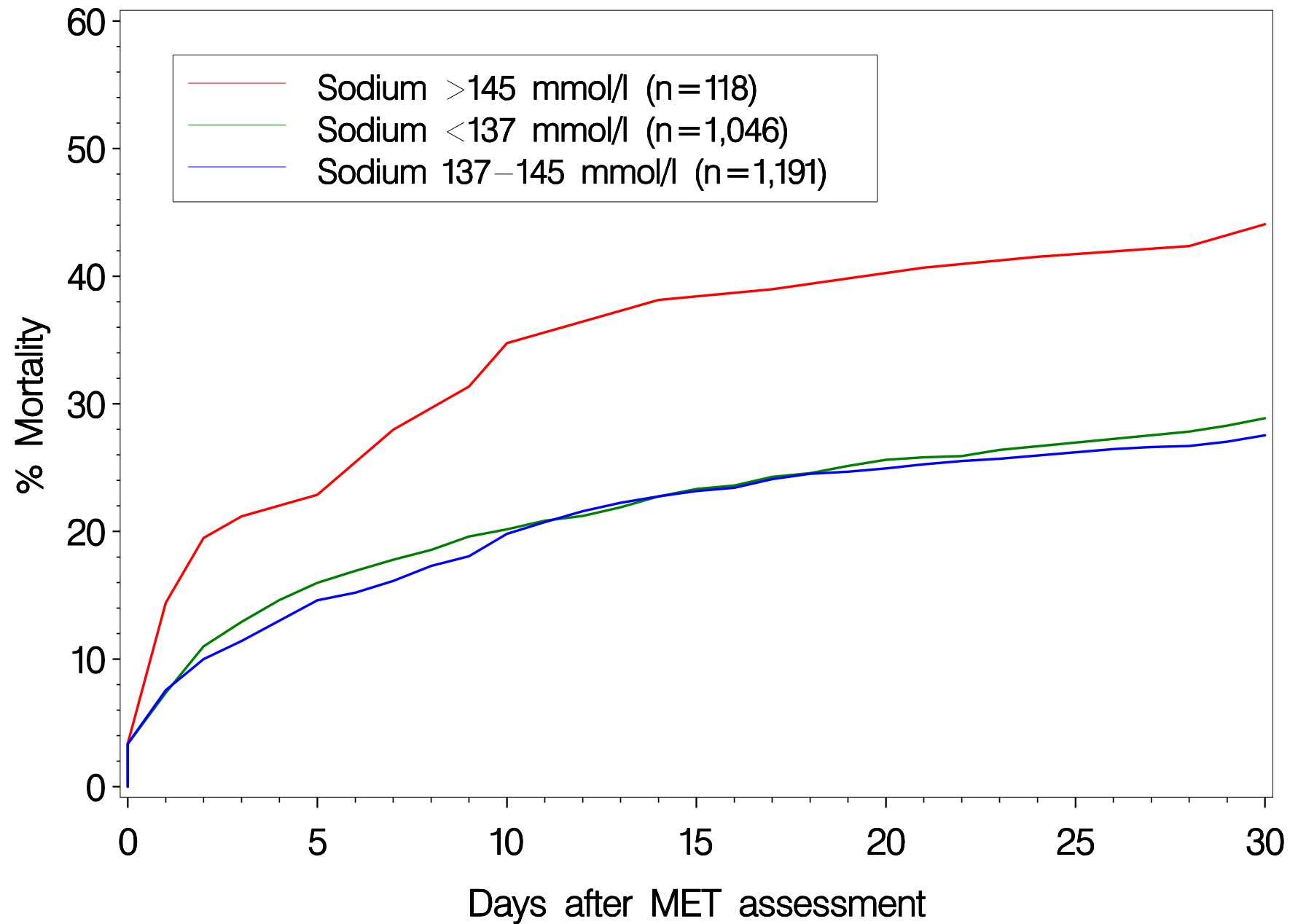

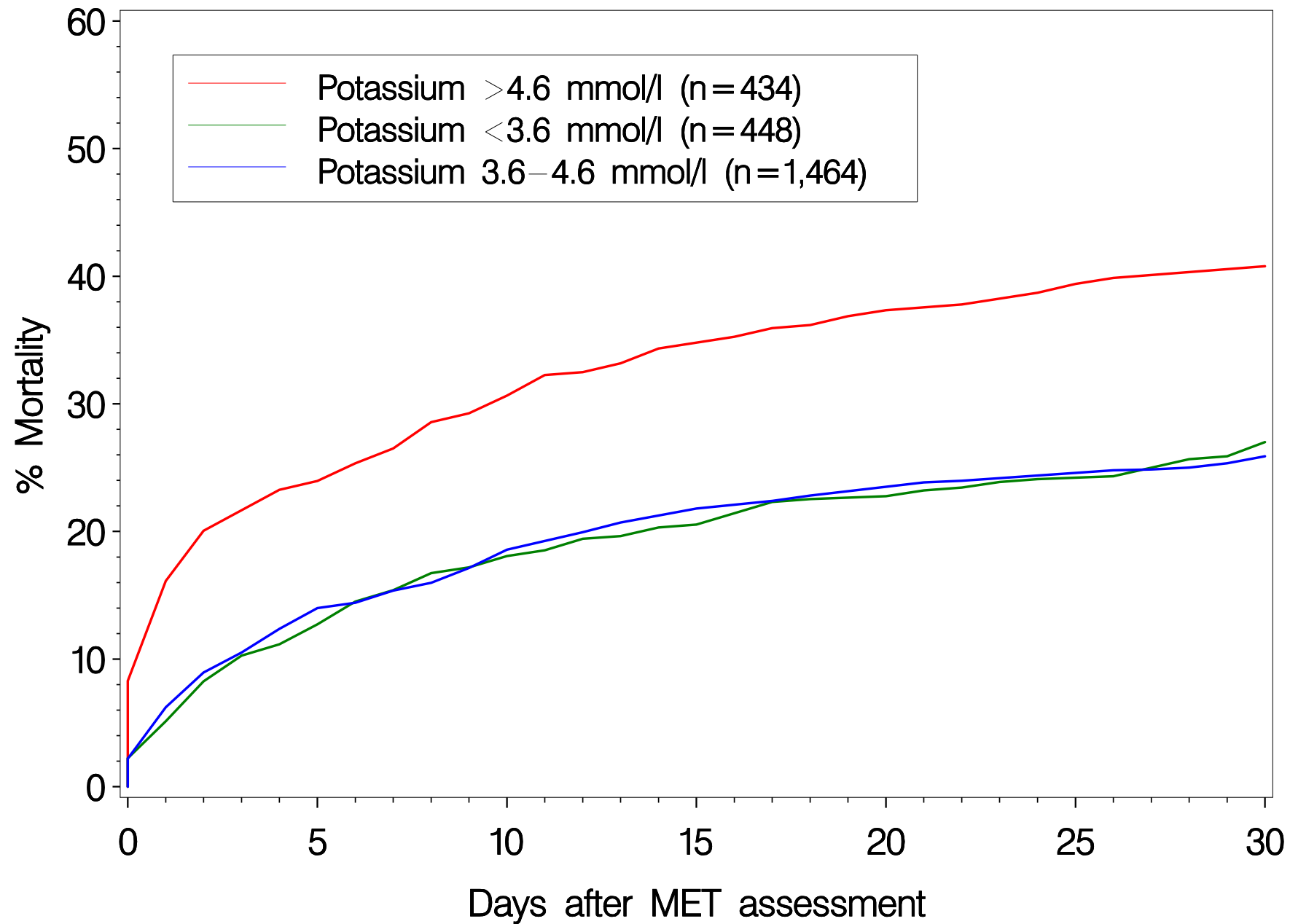

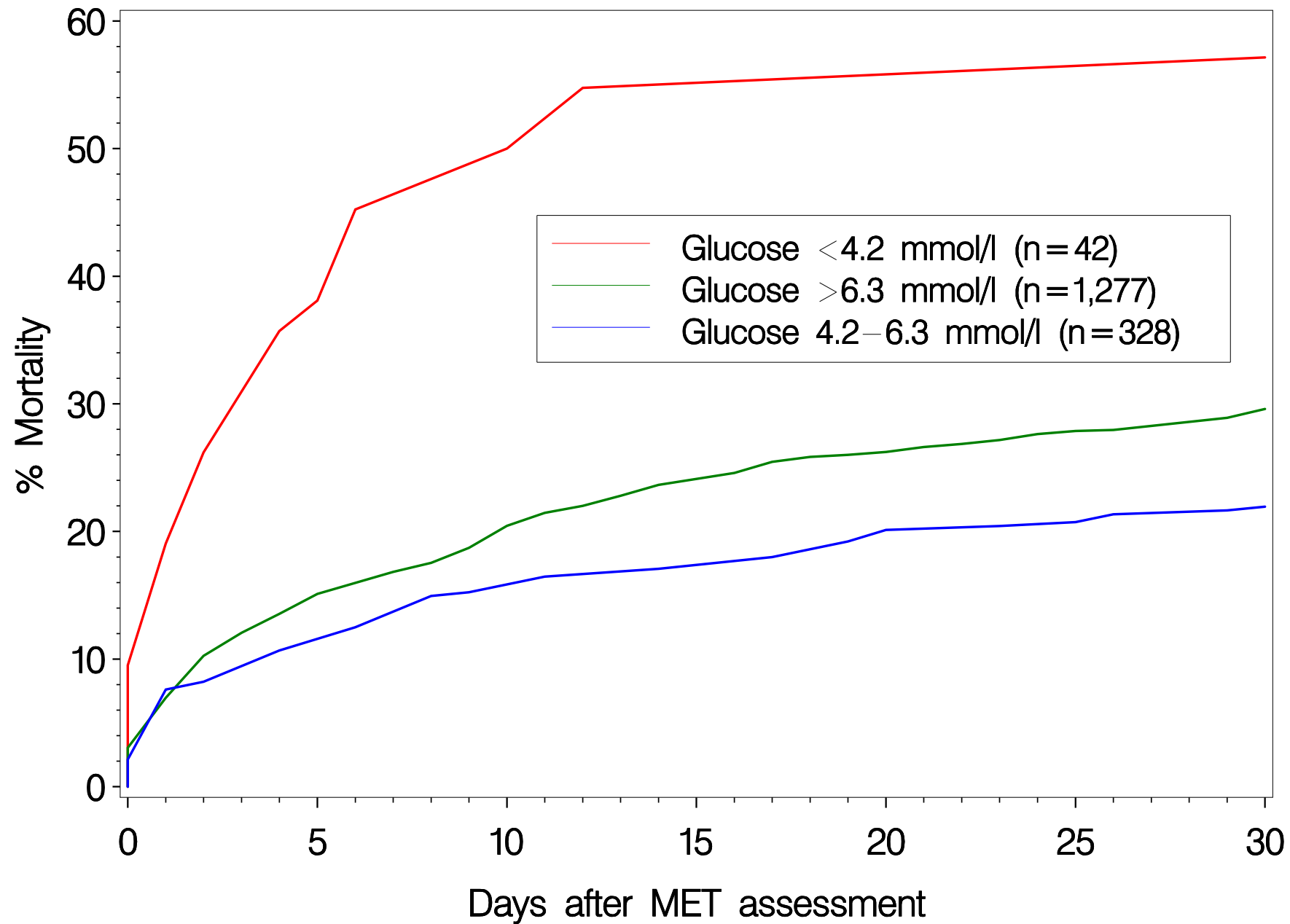

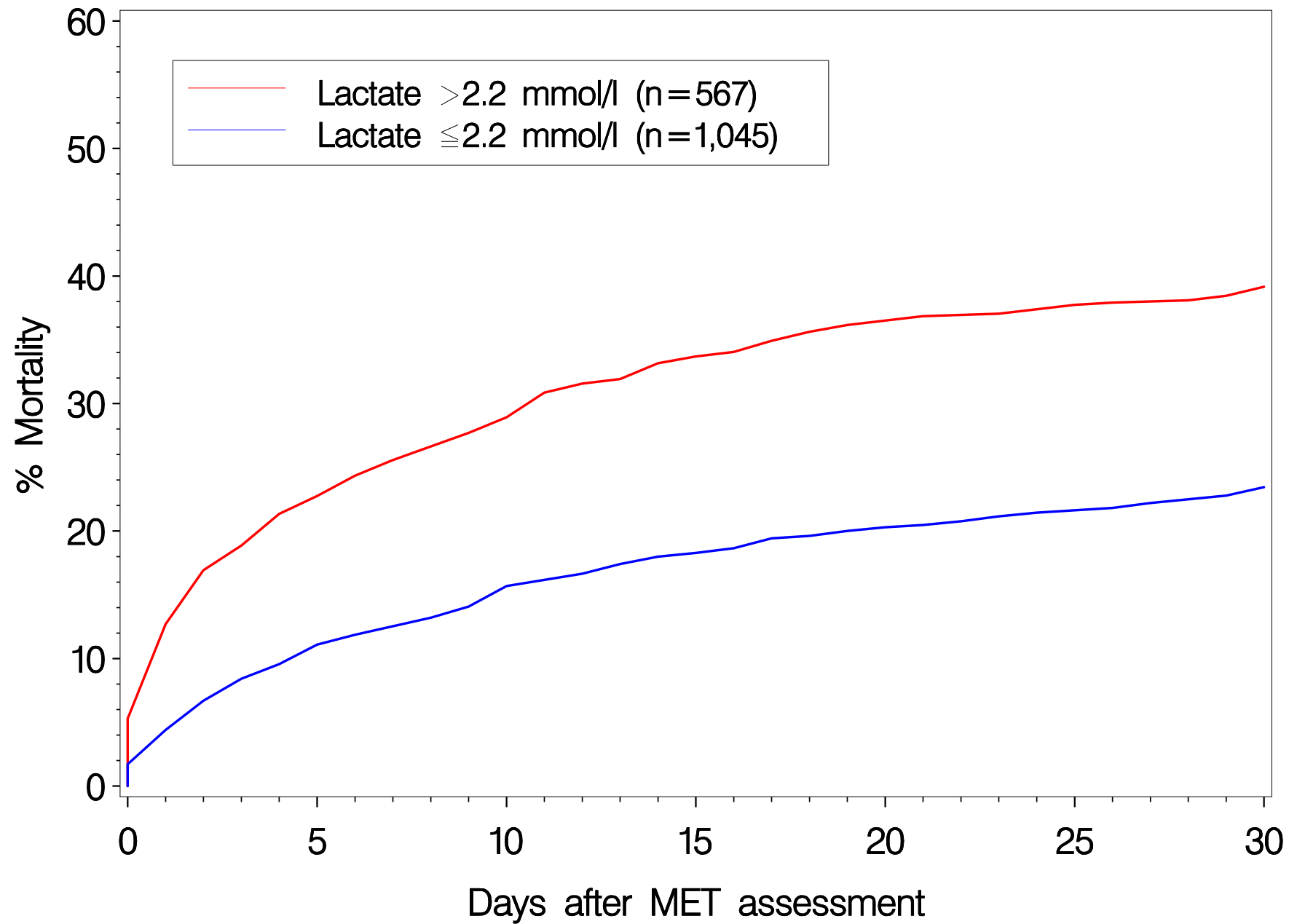

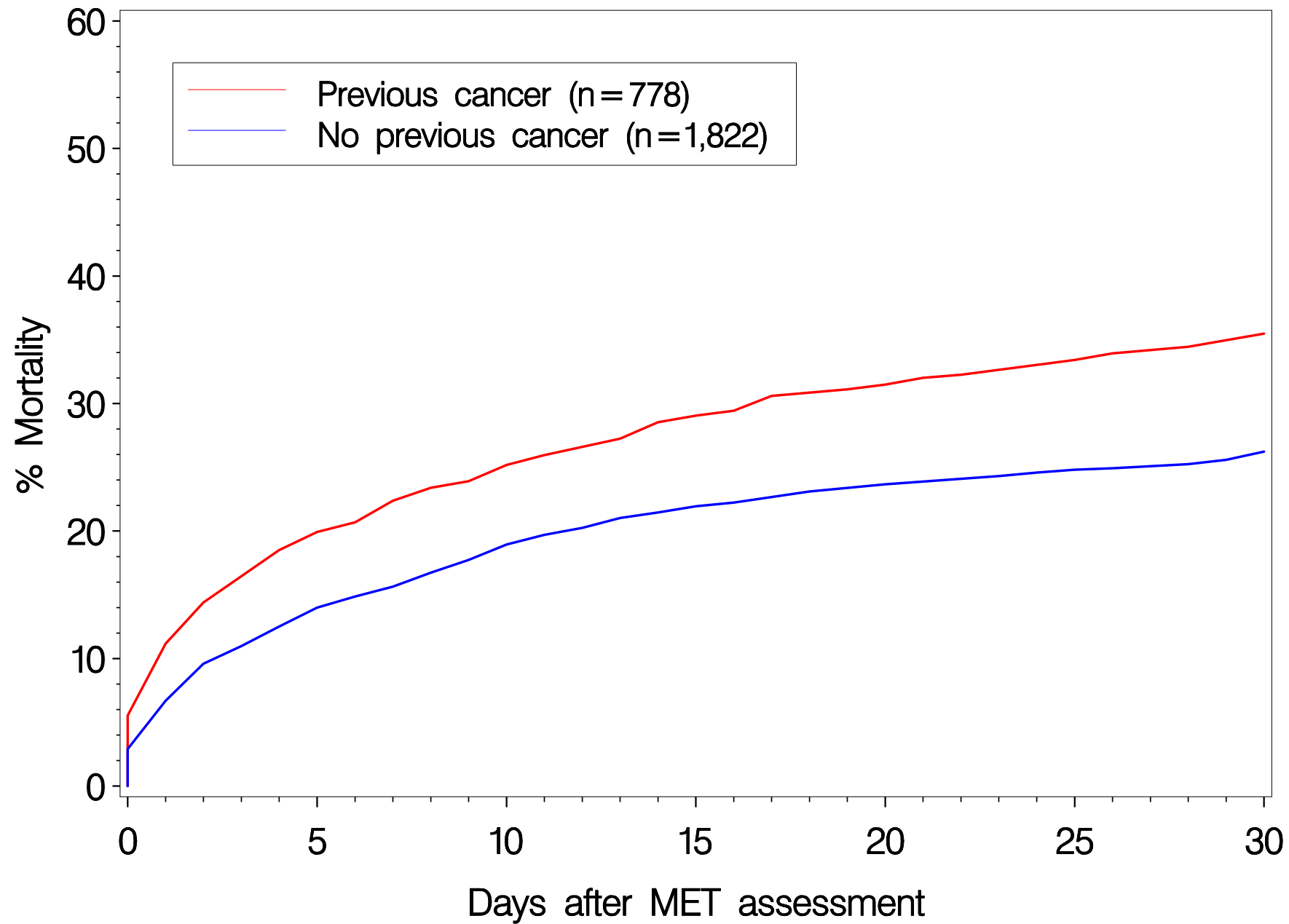

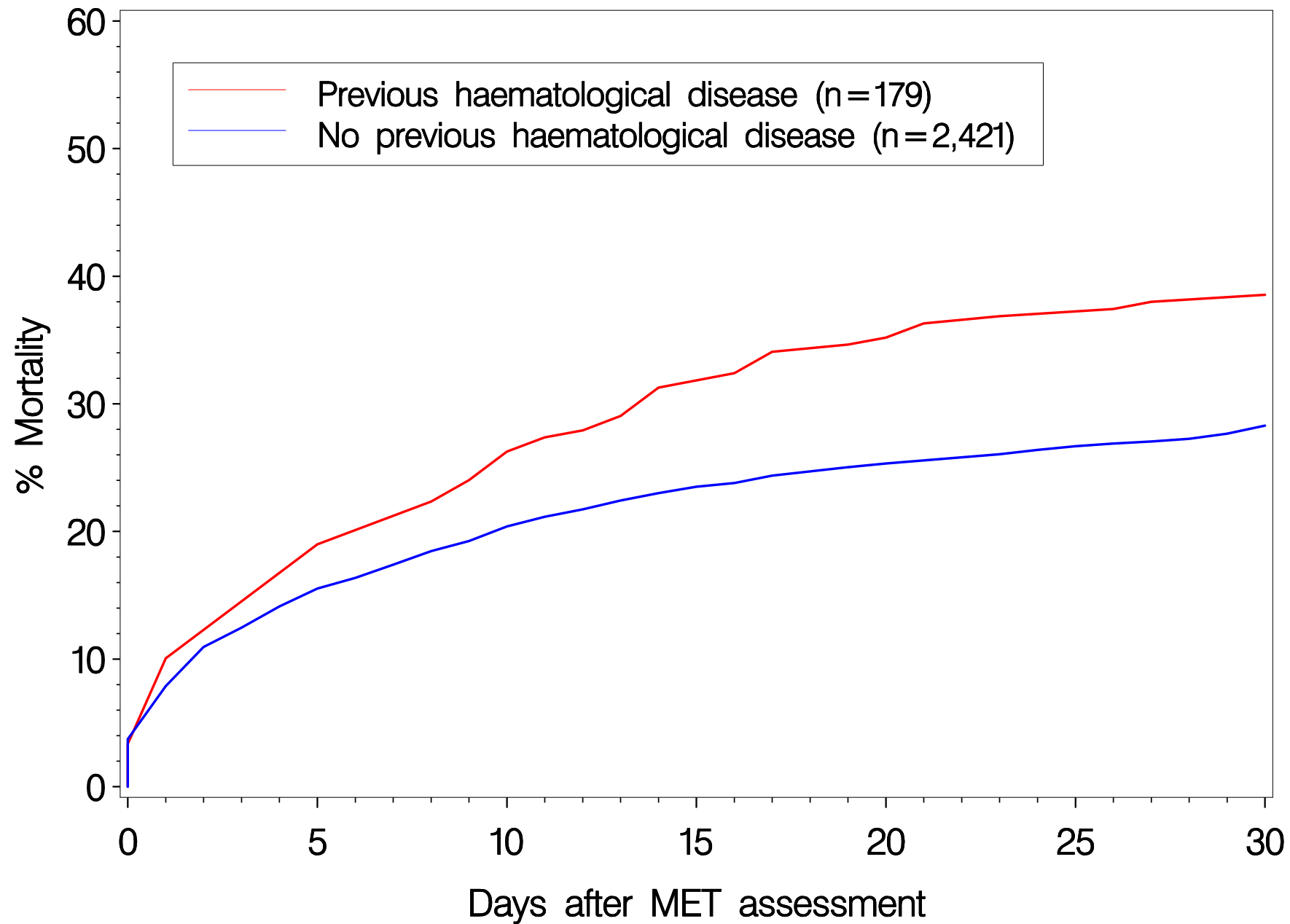

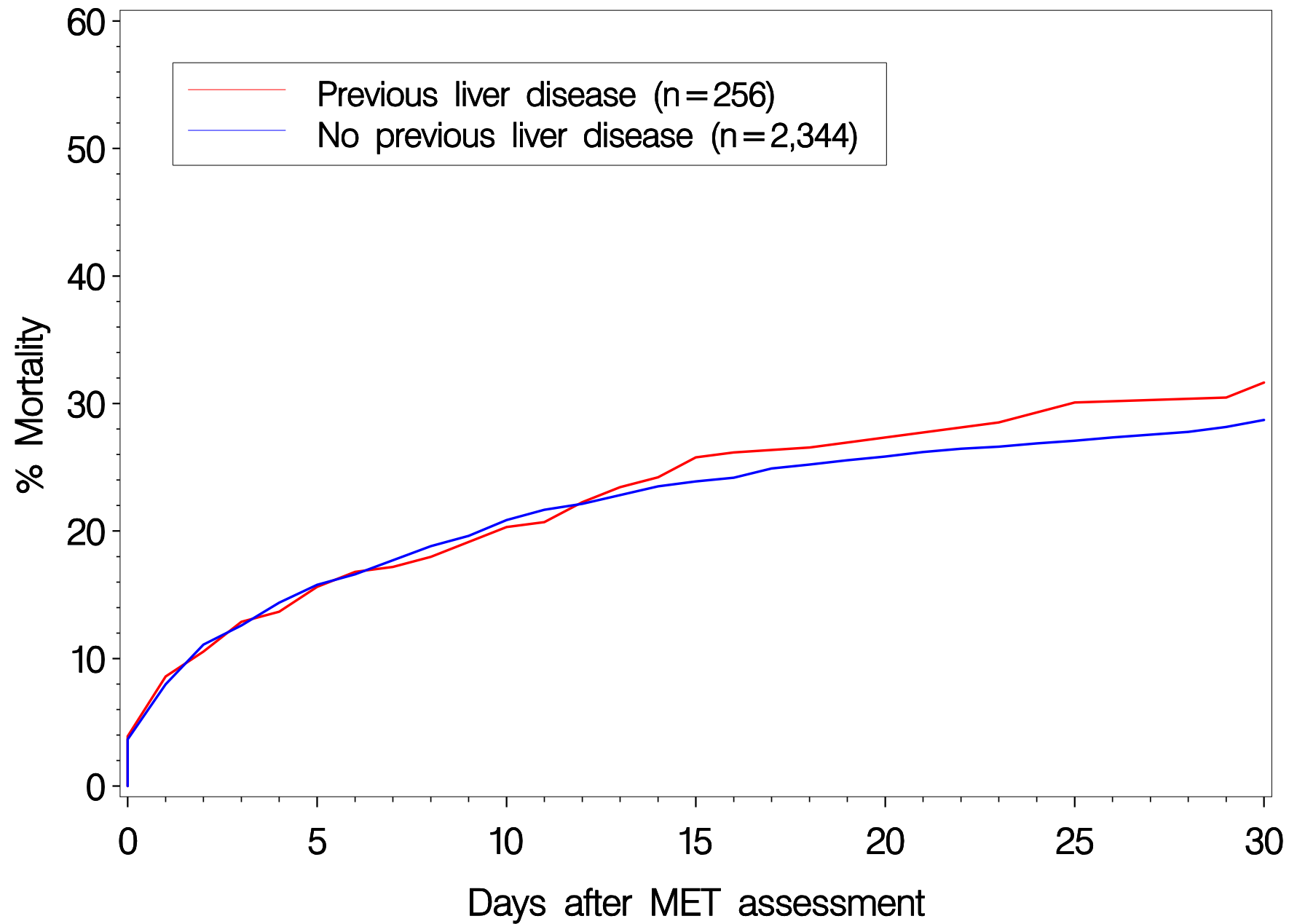

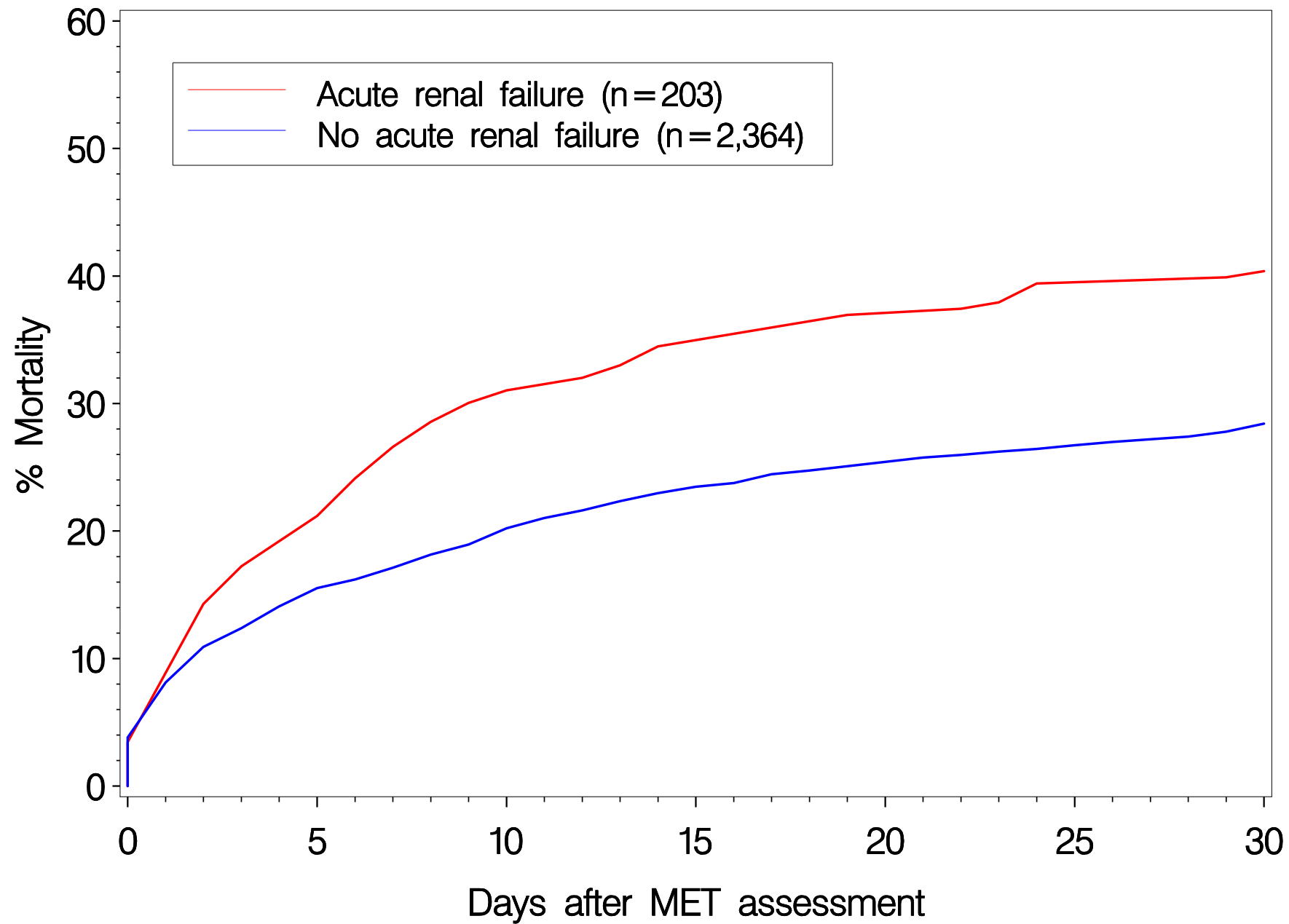

Supplement: Supplementary file 10 — Additional file 10. [file 12873_2022_739_MOESM10_ESM.pdf]
