# Supplementary material for: Outcome prediction for patients assessed by the medical emergency team: a retrospective cohort study
Source: BMC Emerg Med. 2022 Dec 9;22:200. doi: 10.1186/s12873-022-00739-w (PMC9733206; doi:10.1186/s12873-022-00739-w)
Supplement: Supplementary file 11 — Additional file 11. [file 12873_2022_739_MOESM11_ESM.pdf]

Additional file 11

When performing complete data multivariable analysis, age, vital parameters, laboratory biomarkers, type of ward, previous medical history and acute medical condition all contributed to the prediction of mortality. The factors with the highest odds ratio for mortality were hypoglycaemia, haematological disease and renal failure.

MULTIVARIABLE ANALYSIS USING COMPLETE DATA

	**OR (95% CI)**	**p**
AGE (per year)	1.04 (1.03,1.05)	<0.0001
TYPE OF WARD		
Surgical wards	0.40 (0.30,0.54)	<0.0001
VITAL PARAMETERS		
SpO2 <90%	1.84 (1.39,2.43)	<0.0001
RR >30 breaths/min	1.51 (1.14,2.00)	0.004
LABORATORY BIOMARKERS		
Glucose <4.2 mmol/l	4.76 (2.13,10.64)	0.0001
Lactate >2.2 mmol/l	1.56 (1.19,2.05)	0.001
PREVIOUS MEDICAL HISTORY		
Haematological disease	2.27 (1.43,3.61)	0.0005
Cancer	1.63 (1.21,2.18)	0.001
ACUTE MEDICAL CONDITION		
Renal failure	2.08 (1.37,3.15)	0.0006

393 (31.2%) endpoints for 1,260 patients

OR, odds ratio; CI, confidence interval; SpO2, *peripheral capillary oxygen saturation; RR, respiratory rate*

Additional file 11. *Multivariable analysis of factors associated with 30-day mortality for patients where MET was activated while hospitalised in 2010-2015 at Sahlgrenska University Hospital, using complete data*
