# Supplementary material for: Outcome prediction for patients assessed by the medical emergency team: a retrospective cohort study
Source: BMC Emerg Med. 2022 Dec 9;22:200. doi: 10.1186/s12873-022-00739-w (PMC9733206; doi:10.1186/s12873-022-00739-w)
Supplement: Supplementary file 12 — Additional file 12. [file 12873_2022_739_MOESM12_ESM.pdf]

Additional file 12

AGE, GENDER AND PREVIOUS MEDICAL HISTORY IN RELATION TO TYPE OF WARD

	MEDICAL (n=1,141)	SURGICAL (n=1,159)	NEUROLOGICAL (n=284)	PSYCHIATRIC (n=17)
AGE; years (mean/median)	65.3/68	66.4/69	65.2/68	52.5/55
FEMALE GENDER	541 (47.4)	504 (43.5)	99 (34.9)	8 (47.1)
PREVIOUS MEDICAL HISTORY*;				
Myocardial infarction	126 (11.1)	110 (9.5)	19 (6.7)	1 (5.9)
Angina pectoris	161 (14.1)	145 (12.5)	34 (12.0)	2 (11.8)
Cardiac failure	162 (14.2)	110 (9.5)	16 (5.6)	3 (17.6)
Other cardiac diseases**	247 (21.7)	237 (20.4)	61 (21.5)	3 (17.6)
Cardiac arrest	8 (0.7)	11 (0.9)	1 (0.4)	1 (5.9)
Stroke	105 (9.2)	91 (7.9)	52 (18.3)	1 (5.9)
Hypertension	336 (29.5)	366 (31.6)	121 (42.6)	3 (17.6)
Peripheral arterial disease	42 (3.7)	53 (4.6)	10 (3.5)	0 (0.0)
Pulmonary disease	388 (34.0)	190 (16.4)	36 (12.7)	3 (17.6)
Respiratory insufficiency	75 (6.6)	36 (3.1)	8 (2.8)	0 (0.0)
Gastrointestinal disease	98 (8.6)	242 (20.9)	15 (5.3)	2 (11.8)
Liver disease	99 (8.7)	143 (12.3)	9 (3.2)	5 (29.4)
Pancreatic disease	12 (1.1)	54 (4.7)	0 (0.0)	0 (0.0)
Renal disease	123 (10.8)	128 (11.0)	10 (3.5)	0 (0.0)
Endocrine disease	21 (1.8)	22 (1.9)	4 (1.4)	0 (0.0)
Haematological disease	138 (12.1)	33 (2.8)	7 (2.5)	1 (5.9)
Cancer	332 (29.1)	407 (35.1)	38 (13.4)	1 (5.9)
Skeletal disease	144 (12.6)	123 (10.6)	18 (6.3)	2 (11.8)
Rheumatic disease	102 (8.9)	67 (5.8)	17 (6.0)	1 (5.9)
Diabetes	193 (16.9)	190 (16.4)	38 (13.4)	4 (23.5)
Neurological disease	181 (15.9)	183 (15.8)	120 (42.3)	4 (23.5)

Psychiatric disease	60 (5.3)	35 (3.0)	6 (2.1)	13 (76.5)
Addiction	118 (10.4)	98 (8.5)	23 (8.1)	9 (52.9)

** 1 missing for medical ward patients*

*** Including cardiac arrhythmias, valvular heart diseases, pericardial disorders, cardiogenetic disorders or congenital heart defects, among others*

Additional file 12. *Age, gender and previous medical history in relation to type of ward where MET was activated while hospitalised in 2010-2015 at Sahlgrenska University Hospital*
