# Supplementary material for: Outcome prediction for patients assessed by the medical emergency team: a retrospective cohort study
Source: BMC Emerg Med. 2022 Dec 9;22:200. doi: 10.1186/s12873-022-00739-w (PMC9733206; doi:10.1186/s12873-022-00739-w)
Supplement: Supplementary file 13 — Additional file 13. [file 12873_2022_739_MOESM13_ESM.pdf]

Additional file 13

The most frequent reasons for MET activations were hypoxia, hypotension and serious concern on the part of the medical staff. Hypoxia and abnormal tachypnea were associated with a higher 30-day mortality, whereas, in contrast, serious concern on the part of the medical staff was associated with a significantly lower 30-day mortality.

REASON FOR CALL

TRIGGER CRITERIA	DEATH WITHIN 30 DAYS Yes (n=755)	No (n=1,846)	Age adjusted OR (95% CI)	p#
SpO2 <90 %	370 (49.0)	630 (34.1)	1.74 (1.46,2.08)	<0.0001
RR <8 or >30 breaths/min	164 (21.7)	266 (14.4)	1.66 (1.33,2.08)	<0.0001
SBP <90 mmHg	222 (29.4)	572 (31.0)	0.86 (0.71,1.05)	0.13
HR <40 or >130 beats/min	111 (14.7)	289 (15.7)	1.00 (0.78,1.28)	1.00
Decreased consciousness	141 (18.7)	288 (15.6)	1.33 (1.05,1.68)	0.02
Serious concern	143 (18.9)	489 (26.5)	0.69 (0.55,0.86)	0.0007
Threatened airway (288/705)*	37 (7.9)	81 (7.1)	1.12 (0.73,1.72)	0.60

Results presented as number (per cent)
** Number of patients for whom information was missing in the two groups, respectively (criterion removed halfway through the study period)*
# Age-adjusted p-value for association with 30-day mortality

OR, odds ratio; CI, confidence interval; SpO2, peripheral capillary oxygen saturation; RR, respiratory rate; HR, heart rate; SBP, systolic blood pressure

Additional file 13*. Outcome in relation to reason for call where MET was activated for patients while hospitalised in 2010-2015 at Sahlgrenska University Hospital*
